# Adaptive individualized gene pair signatures distinguishing melanoma and predicting response to immune checkpoint blockade

**DOI:** 10.1016/j.isci.2025.113329

**Published:** 2025-08-08

**Authors:** Zhihua Du, Qiyi Chen, Weiliang Huang, Yijun Zhou, Huaijin Wen, Di Wang, Yinghua Chen, Lixin Cheng, Xubin Zheng

**Affiliations:** 1 College of Computer Science and Software Engineering, ShenZhen University, Shenzhen, China; 2 Guangdong Provincial Key Laboratory of Mathematical and Neural Dynamical Systems, School of Computing and Information Technology, Great Bay University, Dongguan, China; 3 Dongguan People’s Hospital Biobank, The Tenth Affiliated Hospital of Southern Medical University, Dongguan, China; 4 Shenzhen People’s Hospital (First Affiliated Hospital of Southern University of Science and Technology, Second Clinical Medicine College of Jinan University), Shenzhen, China

**Keywords:** Oncology, Medical informatics, Artificial intelligence applications

## Abstract

Distinguishing similar cancer subtypes and predicting responses to immune checkpoint blockade (ICB) are critical for improving clinical outcomes. However, existing gene expression signatures often suffer from batch effects and poor generalizability across cohorts. To address these limitations, we propose adaptive individualized gene pair signatures (AIGPS), a robust method that adaptively quantifies gene pair reversals and selects informative features using machine learning. AIGPS was validated on 850 samples from 24 cohorts for multiclass skin cancer classification and on 252 samples from 7 cohorts including both bulk and single-cell RNA sequencing (RNA-seq) data for ICB response prediction in melanoma. Compared to existing approaches, AIGPS improves classification accuracy by over 5% and enhances response prediction performance by 6%. By relying on relative rather than absolute expression levels, AIGPS demonstrates robustness to technical variability and enhanced transferability across datasets. This adaptive framework offers a flexible strategy for biomarker discovery and has broad potential in precision oncology.

## Highlights

• Adaptive individualized gene pair signatures (AIGPS) is proposed
• AIGPS improves melanoma immunotherapy response prediction by 6% over existing methods
• AIGPS outperform differential expressing genes in skin cancer classification

## Graphical abstract

**Figure:**
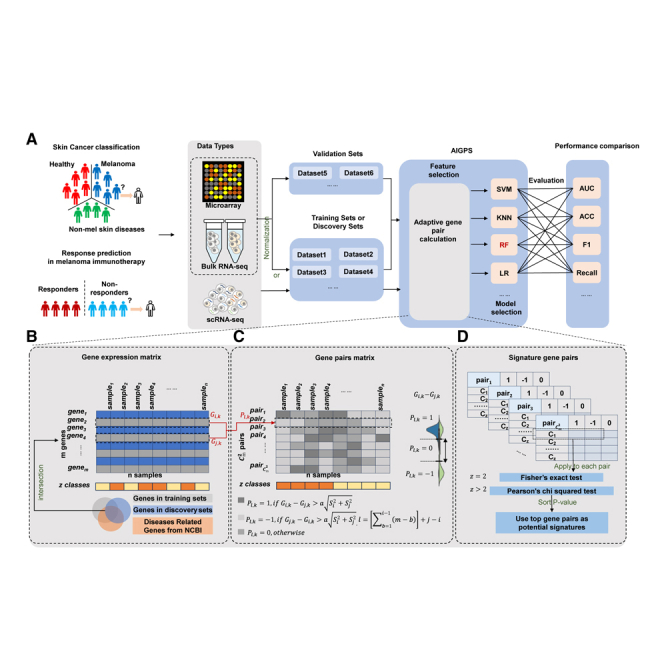

## Introduction

Classification across similar cancer types or multiple pathological progression, such as different types in skin cancer, is significantly important in cancer diagnosis. Melanoma, the deadliest form of skin cancer, is easily confused with other skin cancers at the time of diagnosis. Diagnosis of melanoma involves recognizing changes in moles or skin lesions, with early-stage melanoma being highly treatable through surgery, while metastatic cases pose significant therapeutic challenges.^1^^,^^2^ There is a high degree of biological similarity among different skin pathological states, making the accurate diagnosis of cancer challenging. Most studies have utilized clinical images^3^^,^^4^^,^^5^^,^^6^ or dermoscopic images^7^^,^^8^^,^^9^^,^^10^^,^^11^ methods for skin cancer classification, but transcriptomes can better reflect the pathological alteration in cancer, which would be a better way to distinguish different skin cancers. Gálvez et al. conducted a study using microarray and RNA sequencing (RNA-seq) data to identify reliable skin cancer biomarkers. They found a set of 17 differentially expressed genes (DEGs) that successfully classified seven skin cancer types.^12^ Subsequent studies expanded on this work, discovering a smaller set of eight highly correlated DEGs that effectively identified up to 10 skin pathological cancer types with favorable performance.^13^

Melanoma is characterized by its high metastatic potential and limited response to traditional therapies.^14^^,^^15^^,^^16^ Despite the significant increase in melanoma incidence rate in middle-aged adults from 8.6 per 100,000 person-years in 1970–1979 to 99.1 per 100,000 person-years in 2011–2020,^17^ recent advancements in immunotherapies and targeted therapies have resulted in a slight decrease in mortality rates.^18^ The main difficulties in current treatments include adverse side effects, resistance mechanisms, and the need for more effective therapeutic options to improve patient outcomes.^19^^,^^20^ Fortunately, transcriptome data offers significant advantages in predicting response to immune checkpoint blockade therapies like anti-PD1 blockade.^21^^,^^22^^,^^23^

Previous genomic and transcriptomic studies have proposed biological signatures for predicting the response to immune checkpoint blockade (ICB) therapy in melanoma, including tumor mutational burden (TMB) and neoantigen load,^24^^,^^25^^,^^26^^,^^27^ cytotoxic immune signature (CYT),^28^ neoadjuvant response signature (NRS),^29^ T cell-inflamed genes,^30^ chemokine,^31^ immune cytolytic activity,^32^ immune score,^33^ MHC-I/II,^34^ IMPRES,^35^ and Pathway signatures.^36^ Many of these signatures involve the expression levels of multiple key genes, while IMPRES uniquely focuses on the pairwise transcriptomic relationships among immune checkpoint genes and shows superior performance. However, a study by Carter et al. raised concerns about IMPRES consistency in predicting the response of metastatic melanoma to ICB therapy.^37^ Another signature Pathway represents the pathway scores calculated through single-sample gene set enrichment analysis using gene expression.

While the classification and prediction of transcriptome data using machine learning and deep learning methods has been extensively explored in biological applications,^38^^,^^39^^,^^40^ the inherent fluctuations observed within and across RNA-seq datasets present a significant challenge for the trained classifiers to effectively generalize to external datasets.^41^ Consequently, in the context of multi-cohort analysis, relying solely on gene expression-based key genes as signatures may not lead to satisfactory outcomes. This approach often relies on gene expression matrix normalization or batch effect removal,^42^^,^^43^^,^^44^^,^^45^^,^^46^ which can distort the biological signal or lead to overcorrection.^47^^,^^48^ We previously proposed gene pair analysis based on transcriptome expression data,^49^^,^^50^ which transforms features from gene expression levels to the expression level relationship between two genes. This method demonstrates robustness to different sample distributions, does not necessitate complex normalization, and is less susceptible to noise in single-cell RNA-seq (scRNA-seq) due to its reliance on relative expression between genes.^50^^,^^51^ It can even use scRNA-seq to assist in the classification of bulk data.^50^

However, the analysis of gene pairs only compared the ranking between genes and ignored the quantitative alteration caused by cancer, resulting in the loss of information. Moreover, there are quantitative differences between genes, i.e., some of the genes may have larger variance or fluctuation among individuals. This may lead to poor generalization and performance when using the same scale method for all genes. Therefore, the quantitative alteration in gene pair and the adaptation to genes should be considered.

**Figure 1:**
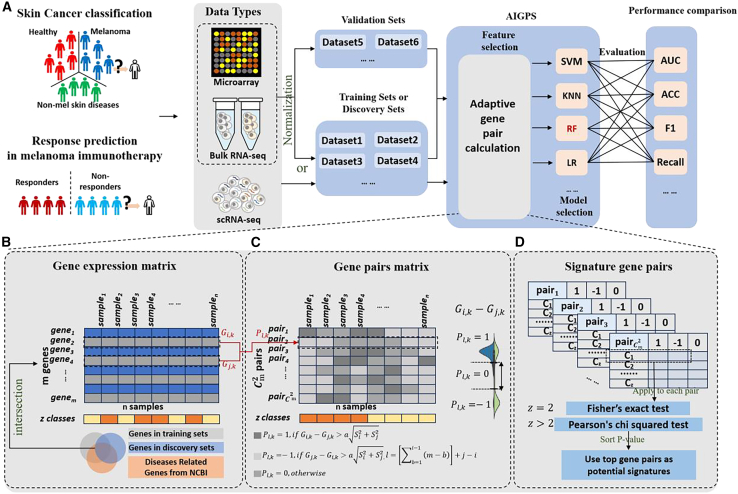
Overview of the study (A) Workflow of the study, encompassing data collection, cohorts partitioning, identification and training of AIGPS, and performance evaluation and comparison. (B) Gene expression matrix and labels obtained from the intersection of multiple cohorts and disease related genes. (C) Exhausted comparison conducted between every two genes within each individual and an adaptive difference derived from the Welch-Satterthwaite’s t test to capture the difference alteration between genes. (D) Gene pairs with reversed expression identified as potential signatures by Fisher’s exact test or Pearson’s chi-squared test. The machine learning models including support vector machine, k-nearest neighborhood, random forest, and logistic regression were applied for the identification of final signature and construction of determination. AIGPS, adaptive individualized gene pair signature; SVM, support vector machine; KNN, k-nearest neighbors; RF, random forest classifier; LR, logistics regression; AUC, area under the curve; ACC, accuracy.

In light of these challenges, we propose the adaptive individualized gene pair signatures (AIGPS) to distinguish melanoma from types of skin cancer and predict response to immune checkpoint blockade in melanoma based on gene expression (Figure 1). Specifically, we transform the gene expression matrix into a gene pair expression matrix and introduce an adaptive difference based on the distribution of two genes within a gene pair in each sample. Gene pairs will be regarded as reversal only when the alteration of the difference between two genes in cancer is larger than the adaptive difference. Subsequently, adaptive reversed gene pairs are screened based on statistical tests, and possibly further screened using random forests to construct AIGPS. In the ICB response prediction, AIGPS were identified from scRNA-seq cohort and trained in two bulk RNA-seq cohorts. The performance of AIGPS with average area under receiver operating characteristic curve (AUC) of 0.68 in four independent test cohorts demonstrates its priority to other ten state-of-the-art methods. In the diagnosis of melanoma in five skin cancer types, we employed 24 microarray cohorts for training and testing, and the F1 score of AIGPS achieved 0.96, all ahead of the published signatures. There are several contribution of AIGPS: (1) introducing adaptive quantification of difference alteration in gene pair, which preserves quantitative information for rank-based methods; (2) constructing gene pair signature to distinguish different skin cancer types and outperforming state-of-the-art biomarkers; (3) constructing gene pair signature to predict ICB response for melanoma prior to state-of-the-art biomarker; (4) better generalization ability between cohorts and capable to concatenate scRNA-seq and bulk RNA-seq data because the relative expressions of gene pairs are less affected by technical variations; (5) better explainability than deep learning methods. In conclusion, AIGPS demonstrates a strong ability to generalize across different datasets and shows promise as a predictive method not only in melanoma but also in other diseases.

## Results

### Extracting potential adaptive gene pairs from scRNA-seq in immunotherapy response

**Figure 2:**
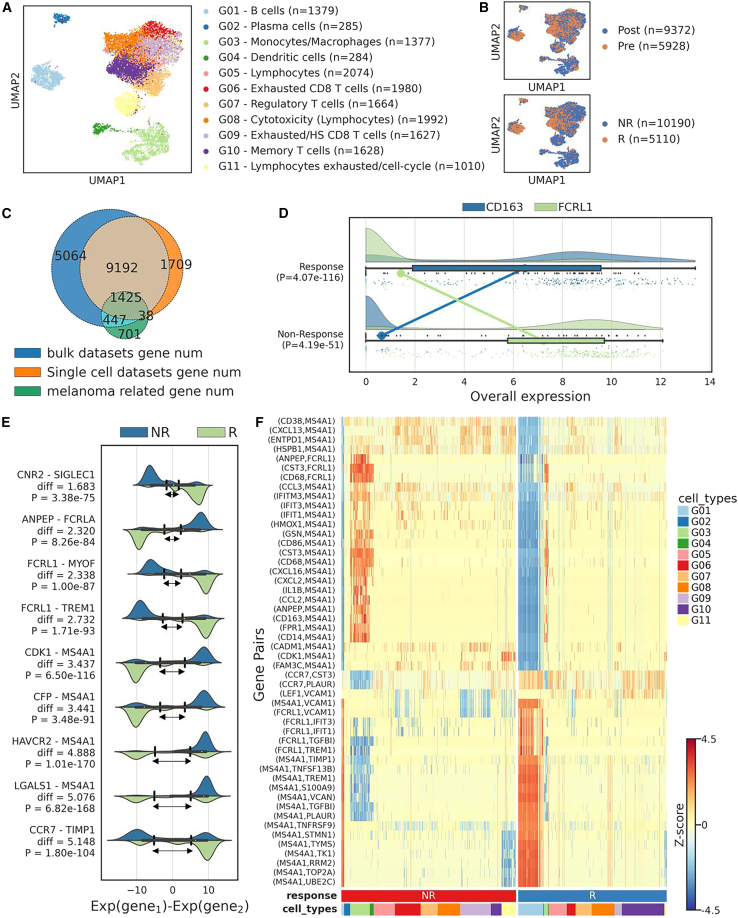
Identification of reversed adaptive gene pairs to predict the response of immune checkpoint blockade on single-cell RNA-seq profile (A) Umap visualization of eleven distinct cell types. (B) Umap visualization of cells collected from pre- and post-treatment samples or responsive and non-responsive samples. (C) Venn diagram of genes among multiple cohorts and melanoma-related genes obtained from NCBI. (D) Expression levels of CD163 and FCRL1 in responsive and non-responsive cells, with corresponding *p* values calculated using Welch’s t test. (E) Alterations of expression differences in reversed gene pairs between responsive and non-responsive cells. *p* values obtained through Pearson’s correlation analysis. (F) Heatmap illustrating the differences of 50 reversed gene pairs identified in AIGPS-50 across different cell types and response statuses.

To identify AIGPS that can predict patients’ response to ICB in the treatment of melanoma, we collected one scRNA-seq cohort and 6 RNA-seq cohorts of pre-treatment melanoma biopsies (Table 1). Due to the limited number of bulk samples, we utilized the scRNA-seq cohort GSE120575,^52^ which involved 48 melanoma patients treated by checkpoint inhibitors, as the discovery set to find potential adaptive gene pairs. The tumor samples in GSE120575 were collected from 32 patients, resulting in a total of 15,300 cells. Using k-means clustering, we identified 11 distinct cell subtypes annotated by the proposed markers in previous study^52^ (Figure 2A). These cells were also labeled as pre-treatment cells (*n* = 5,928) and post-treatment cells (*n* = 9,372) or as response cells (*n* = 5,110) and non-response cells (*n* = 10,190; Figure 2B). Moreover, we took intersection and obtained 1,425 common genes among single-cell cohort, bulk RNA cohorts, and melanoma-related genes obtained from NCBI^53^ (Figure 2C).

**Table 1:** Cohorts collected to identified AIGPS in predicting response of immune checkpoint blockade in melanoma

Cohort	Technology	Sample preservation methods	Pre-treatments samples (cells)			Treatment	Reference
			Response	Non-response	Counts		
GSE120575	scRNA-seq	Fresh tissue	2725	3203	5928	PD1, CTLA4+PD1, CTLA4 (baseline); PD1 (post I and II), CTLA4 (baseline); PD1 (post I)	Sade-Feldman et al.
Riaz	RNA-seq	FFPE, fresh tissue	18	31	49	Anti-PD1 without previous anti-CTLA4, Anti-PD1 with previous anti-CTLA4	Riaz et al.
Van	RNA-seq	FFPE	12	29	41	Anti-CTLA4 monotherapy	Van et al.
Hugo	RNA-seq	–	15	12	27	Anti-PD1 monotherapy	Hugo et al.
Lee	RNA-seq	FFPE	22	22	44	Anti-PD1 monotherapy	Lee et al.
Gide	RNA-seq	FFPE	45	27	72	Anti-PD1 monotherapy, Anti-PD1 + anti-CTLA4 treatment	Gide et al.
MGH	RNA-seq	FFPE, fresh tissue	6	13	19	Anti-PD1 monotherapy, Anti-PDL1 monotherapy, Anti-PD1+anti-CTLA4	Auslander et al.
Bulk Total			**118**	**134**	**252**

We conducted a screening of adaptive reversed gene pairs within the single-cell discovery set and identified 100 pairs for further analysis (refer to the STAR Methods section). As an illustrative example, we examined *CD163-FCRL1* and observed notable differences in expression patterns. Specifically, *CD163* exhibited relatively higher expression level in responsive cells (*p* = 4.07e-116; Welch’s t test), while FCRL1 showed lower expression levels. Conversely, in non-responsive cells, *CD163* displayed lower expression levels and FCRL1 showed higher expression levels (*p* = 4.19e-51; Welch’s t test; Figure 2D). These findings suggest a potential association between the expression levels of *CD163* and *FCRL1* and the responsiveness of patients to ICB.

To demonstrate the adaptability of AIGPS, we depicted the adaptive variances of gene pairs in Figure 2E. As the difference alterations of each gene pair increase within responsive samples, the adaptive difference range also expands, which consistently falls within the range between the two peaks of the gene pair difference distribution. Moreover, we calculated the Pearson’s correlation and found the association between the gene pair differences in each cell and the corresponding treatment label (response or non-response). This analysis emphasizes the role of adaptive differences in the comparison of gene pair expressions to gain a deeper understanding of their potential impact on treatment response.

We also presented the expression difference of the top 50 adaptive gene pairs in each cell and observed that the most pairs exhibited larger reversal differences of expression in G01 (B cells) and G03 (monocytes/macrophages), demonstrating that B cells and monocytes/macrophages are possible determinant in the ICB response (Figure 2F). This finding also suggests that the AIGPS can reflect the distinctions between certain cell types from other aspect.

**Figure 3:**
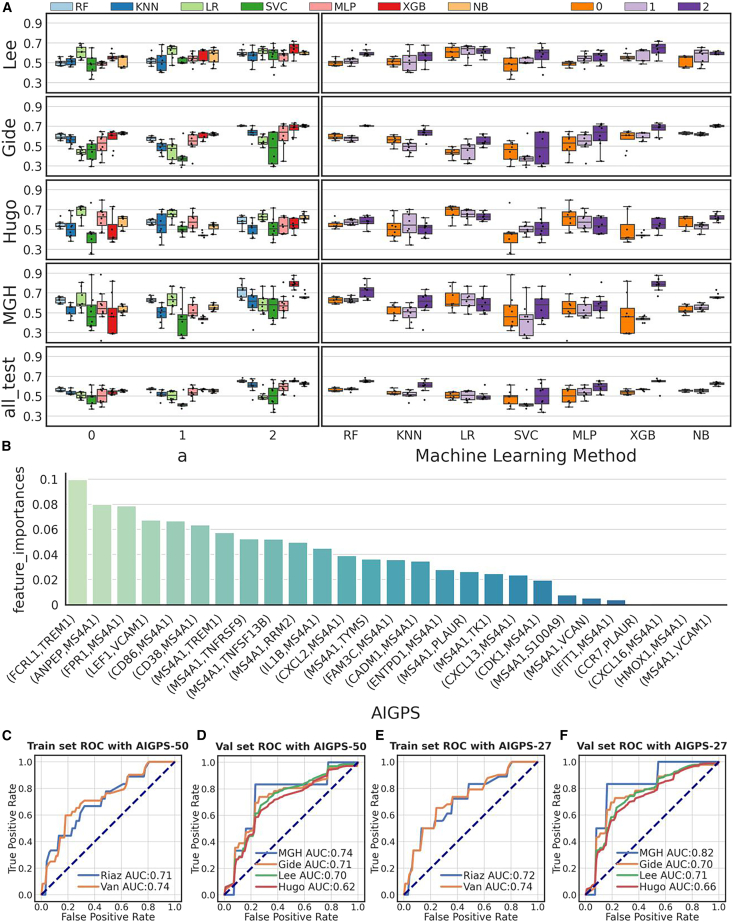
Construction of AIGPS predicting response of immune checkpoint blockade (A) Boxplot displaying the performance of different adaptive coefficients and machine learning methods on independent validation cohorts, data are represented as mean ± SEM. (B) Barplot of the non-zero feature importances in AIGPS-50 in predicting immune checkpoint blockade (ICB) response. (C) Performance of AIGPS-50 in the training sets. (D) Performance of AIGPS-50 in validation sets. (E) Performance of AIGPS-27 in the training sets. (F) Performance of AIGPS-27 in validation sets. RF, random forest classifier; KNN, k-nearest neighbors; LR, logistics regression; SVM, support vector machine; MLP, multi-layer perception; XGB, extreme gradient boosting; NB, naive bayes; ROC, receiver operating characteristic.

Construction of AIGPS in immunotherapy response prediction. To obtain the best performance of AIGPS, we compared different adaptive coefficients and machine learning methods including random forest classifier (RF), k-nearest neighbors (KNN), logistics regression (LR), support vector machine (SVM), multi-layer perception (MLP), extreme gradient boosting (XGB), and naive Bayes (NB), with 3-fold cross-validation and AUC as evaluation (Figure 3A). To optimize AIGPS performance, we evaluated adaptive coefficients across a range of values (from zero to five with 0.5 increments). Sensitivity analysis demonstrated that a = 2.0 maximized median AUC while minimizing variance across most immunotherapy cohorts, with consistent results in independent datasets using RF, SVM, and other classifiers (Figure S2). When comparing different machine learning methods, the random forest classifier (RF) demonstrated prior performance and greater stability compared to other methods. Therefore, random forest was applied as the discriminative model. Furthermore, we found that a set of 50 adaptive gene pairs with the random forest classifier achieved better performance (Figure S3). Specifically, the AUC of these 50 pairs (AIGPS-50) for the Riaz cohort and Van cohort as training sets reached 0.71 and 0.74, respectively (Figure 3C). In the independent test cohorts, the AUC values for the MGH cohort, Gide cohort, Lee cohort, and Hugo cohort reached 0.74, 0.71, 0.70, and 0.62, respectively (Figure 3D). Additionally, AIGPS-50 achieved AUC values of 0.71 and 0.69, respectively, in the combined training set and test set when combining the cohorts together (Figures S4A and S4B). We further conducted a residual validation, and the results also demonstrated the robustness of AIGPS (Figure S5).

We also attempted to adopt other screening strategies, such as the LASSO feature screening method. However, directly using LASSO regression would consume excessive computing resources. Therefore, we first screened out 100,000 pairs of genes based on *p* values and then used LASSO to select 138 pairs of genes with non-zero coefficients from them. Subsequently, we sorted by coefficient size and successively selected the top 10 to 130 pairs of genes for model training and testing. The results show that the gene pairs screened based on *p* value have a better predictive effect compared with those screened by LASSO (Figure S6).

We also compared the performance of different numbers of highly variable genes and melanoma-related genes (Figure S7). Under different adaptive coefficients, 50 gene pairs were selected for 10 repeated experiments, and the results showed that the performance of related genes was better than that of highly variable genes. Moreover, including single-cell data were far ahead of the results using only bulk data in both settings. To prevent overfitting during model training, we adopted the three-way cross-validation method throughout the process and applied SMOTE balance to the training set to handle the problem of class imbalance. However, the obtained effect was not as good as not using SMOTE (Figure S8).

To obtain the best combination of gene pairs, we calculated the feature importance of gene pairs in RF and removed pairs with a feature importance of 0 (Figure S9), resulting in a final set of 27 adaptive reversed gene pairs (Figure 3B). Next, we utilized these pairs to train a random forest model and constructed the discriminative model named AIGPS-27. Evaluation of AIGPS-27 demonstrates a prior performance to AIGPS-50. The AIGPS-27 achieved AUCs of 0.72 and 0.74 in the training Riaz cohort and Van cohort, respectively (Figure 3E) and AUC of 0.72 for the combined training set (Figure S10A). In terms of the test set, the AUC of AIGPS-27 in the MGH cohort, Gide cohort, Lee cohort, and Hugo cohort are 0.82, 0.70, 0.71, and 0.66, respectively (Figure 3F), and the AUC in the combined test set is 0.69 (Figure S10B). The performance metrics of the AIGPS-27 model across the test cohorts, including negative predictive value (NPV), positive predictive value (PPV), accuracy, AUC, balanced accuracy, F1-score, recall, and specificity, are comprehensively detailed in Table S2.

Among the reversed gene pairs in AIGPS-27, *FCRL1-TREM1* exhibited the highest importance. *FCRL1* encodes a member of the immunoglobulin receptor superfamily and has been identified as a potential biomarker for prognosis and a therapeutic target in diffuse large B cell lymphoma (DLBCL) treatment.^54^ *TREM1* is also a crucial receptor involved in immune responses and inflammation.^55^^,^^56^

### Performance of AIGPS-27 in ICB response prediction of melanoma

**Figure 4:**
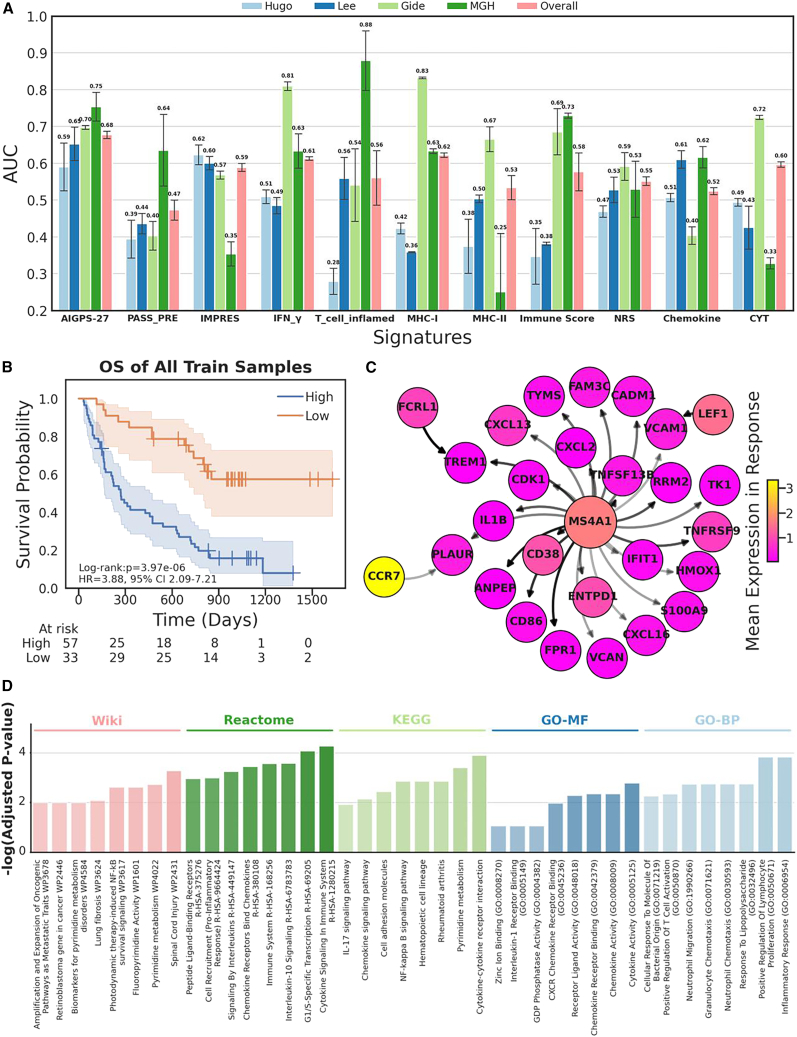
Comparison and analysis of AIGPS (A) The performance of AIGPS-27 and the compared existing signatures in independent validation cohorts, data are represented as mean ± SEM. (B) Kaplan-Meier survival curve of samples in the entire training cohort, where patients are divided into two groups based on the mean odds ratio of AIGPS determinant model. Statistical analysis is conducted using a two-sided log rank test, and the hazard ratio and 95% confidence interval are provided. (C) Gene network constructed by AIGPS-27. The arrows on the edges indicate that the mean expression of gene A is higher than that of gene B in cells from responsive patients. The transparency of the edge represents the feature importance of the pairs in the random forest algorithm, and the size of the node represents the degree. (D) Enrichment analysis of the genes involved in AIGPS-27. The top 8 enriched pathways with adjusted *p* value less than 0.05 are displayed in Gene Ontology Cellular Component (GO-CC), Gene Ontology Biological Process (GO-BP), Gene Ontology Molecular Function (GO-MF), KEGG Pathway, Reactome Pathway, and Wiki Pathway.

We compared AIGPS-27 to existing transcriptome-based predictive signatures including PASS-PRE signatures,^36^ IMPRES signatures,^35^ IFN-γ signatures,^30^ T cell inflamed signatures,^30^ MHC-I,^34^ MHC-II,^34^ Immune Score,^33^ NRS,^29^ Chemokine,^31^ and CYT^28^ (Table S1). The mean AUC of AIGPS with 3-fold cross-validation in Hugo, Lee, Gide, MGH, and the overall cohorts is 0.59, 0.65, 0.70, 0.75, and 0.68, respectively, demonstrating that the overall performance of AIGPS-27 is better than other signatures (Figure 4A). Although the T cell inflamed signatures demonstrated superior performance compared to our approach on the MGH cohort, and IFN-γ signatures and MHC-I showed better performance on the Gide cohort, they exhibited significant poor performance on the remaining cohorts. These results also demonstrated the generalizability of AIGPS-27 in multiple cohorts.

### Survival analysis of AIGPS in immunotherapy

To explore the association between response to ICB and the survival of patients, we conducted survival analysis of AIGPS. We applied the odds ratio obtained by the score from AIGPS to divide the sample into low-risk group and high-risk group. In all training patients, we observed that the low-risk group survived significantly longer than the high-risk group (Kaplan-Meier log rank test: *p* = 3.97e-6, hazard ratio [HR] = 3.88, 95% confidence interval [CI]: 2.09–7.21; Figure 4B).

When examining the Riaz cohort and Van cohort in the training set, we found consistent results regarding overall survival (OS) and progression-free survival (PFS). The Riaz cohort demonstrated significant differences in OS (Kaplan-Meier log rank test *p* = 0.004, HR = 3.24, 95% CI: 1.40–7.51) and a trend toward significance in PFS (Kaplan-Meier log rank test *p* = 0.06, HR = 1.86, 95% CI: 0.97–3.56). Similarly, the Van cohort showed significant differences in both OS (Kaplan-Meier log rank test *p* = 4.51e-4, HR = 4.2, 95% CI: 1.77–9.97) and PFS (Kaplan-Meier log rank test *p* = 0.006, HR = 2.65, 95% CI: 1.29–5.45; Figures S11A–S11D). These findings indicate that the predicted risk groups based on AIGPS can effectively stratify patients and correlate with their survival outcomes.

### Biological functions of AIGPS in immunotherapy

To evaluate the effectiveness of the identified AIGPS-27, gene pairs were utilized to construct gene network in order to identify key genes within the network. Gene set enrichment analysis (GSEA) was then employed to explore the functions associated with these genes.

In the gene network of AIGPS-27, each edge connects to two genes in a pair (Figure 4C). Among these genes, *MS4A1* is connected to the majority of other genes, indicating its central position in the gene network. *MS4A1* exhibits a high expression level in response cells, suggesting its significant role in immunotherapy.

The genes from AIGPS-27 are subjected to GSEA using various databases, such as KEGG, Reactome, Wiki, and gene ontology. The results of GSEA reveal that these genes are closely associated with terms, such as cytokine activity, inflammatory response, cytokine signaling in immune system, cytokine-cytokine receptor interaction, and spinal cord injury (Figure 4D). To complement these findings, we systematically summarize the core biological evidence for all 27 AIGPS-identified gene pairs, including their experimentally validated immune or melanoma-related functions, literature citations, and enriched signaling pathways overlapping with the GSEA results (Table S3). These genes enrich in functions related to the immune system, which provides an explanation for the effectiveness of AIGPS. This indicates that the gene pairs identified by AIGPS are involved in cytokine activity, inflammatory responses, cytokine signaling in the immune system, and immune-related processes. These findings further support the notion that AIGPS is capturing relevant gene interactions and highlighting the importance of the immune system in the context of the analyzed data.

### AIGPS in skin cancer diagnosis

To accurately distinguish different skin cancer types, a comprehensive microarray profile of skin tissue was obtained. This dataset consists of 850 samples, encompassing five different statuses: healthy samples, squamous cell carcinoma (SCC), basal cell carcinoma (BCC), Merkel cell carcinoma (MCC), and melanoma (MEL) (Table 2).

**Table 2:** Cohorts collected to construct AIGPS for skin cancer classification

Datasets	Platform	Technology	Sample preservation methods	Annotation data chip	Healthy	BCC^a^	SCC^b^	MCC^c^	MEL^d^	Count
GSE02503	GPL96	Affymetrix	Fresh tissue	hgu133a.db	6		5			11
GSE03189	GPL96	Affymetrix	–	hgu133a.db	25				45	70
GSE06710	GPL96	Affymetrix	Fresh tissue	hgu133a.db	13					13
GSE07553	GPL570	Affymetrix	Fresh tissue	hgu133plus2.db	4	15	11		56	86
GSE13355	GPL570	Affymetrix	Fresh tissue	hgu133plus2.db	64					64
GSE14905	GPL570	Affymetrix	Fresh tissue	hgu133plus2.db	21					21
GSE15605	GPL570	Affymetrix	Fresh tissue	hgu133plus2.db	16				58	74
GSE29359	GPL6104	Illumina	Fresh tissue	illuminaHumanv2.db					82	82
GSE30999	GPL570	Affymetrix	Fresh tissue	hgu133plus2.db	85					85
GSE32407	GPL571	Affymetrix	Fresh tissue	hgu133a2.db	10					10
GSE32628	GPL6102	Illumina	Fresh tissue	lumiHumanAll.db			15			15
GSE32924	GPL570	Affymetrix	Fresh tissue	hgu133plus2.db	8					8
GSE36150	GPL5175	Affymetrix	FFPE	huex10sttranscriptcluster.db				15		15
GSE39612	GPL570	Affymetrix	Fresh tissue	hgu133plus2.db		2	4	30		36
GSE42109	GPL570,GPL571	Affymetrix	Fresh tissue	hgu133a2.db		11				11
GSE42677	GPL571	Affymetrix	Fresh tissue	hgu133a2.db/hgu133plus2.db	10		10			20
GSE45216	GPL570	Affymetrix	Fresh tissue	hgu133plus2.db			30			30
GSE46517	GPL96	Affymetrix	Fresh tissue	hgu133a.db	16				83	99
GSE50451	GPL570,GPL571	Affymetrix	Fresh tissue	hgu133plus2.db				23		23
GSE52471	GPL571	Affymetrix	Fresh tissue	hgu133a2.db	13					13
GSE53223	GPL570	Affymetrix	Fresh tissue	hgu133plus2.db	18					18
GSE53462	GPL10558	Illumina	Fresh tissue	lumiHumanAll.db	5	16	5			26
GSE66359	GPL570	Affymetrix	Fresh tissue	hgu133plus2.db			8			8
GSE82105	GPL570	Affymetrix	–	hgu133plus2.db	6				6	12
**Total**					**320**	**44**	**88**	**68**	**330**	**850**

a BCC, Basal cell carcinoma.

b SCC, Squamous cell carcinoma.

c MCC, Merkel cell carcinoma.

d MEL, melanoma.

**Figure 5:**
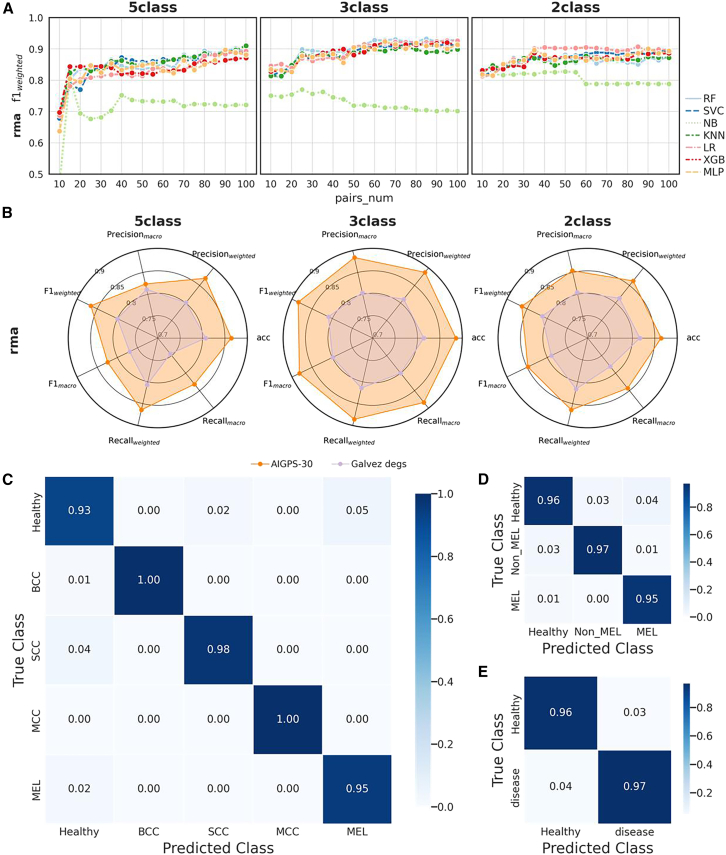
AIGPS in skin cancer diagnosis (A) The weighted F1-score of various machine learning methods with different numbers of reversed adaptive gene pairs on independent validation cohorts. (B) Performance of AIGPS comparing to Galvez’s differentially expression genes in two-class classification (healthy and disease), three-class classification (healthy, Non-MEL, and MEL), and five-class classification (healthy, BCC, SCC, MCC, and MEL) on independent validation cohorts. (C–E) The performance of AIGPS in each categories of 5-class (C), 2-class (D), and 3-class (E) classification. RF, random forest classifier; KNN, k-nearest neighbors; LR, logistics regression; SVC, support vector classifier; MLP, multi-layer perception; XGB, extreme gradient boosting; NB, naive bayes; ROC, receiver operating characteristic; BCC, Basal cell carcinoma; SCC, squamous cell carcinoma; MCC, Merkel cell carcinoma; MEL, melanoma.

For the collected samples, we examined AIGPS in three classification tasks, including two categories (healthy and disease), three categories (healthy, Non-MEL, and MEL), and five categories (healthy, BCC, SCC, MCC, and MEL) classification (Table 3). Following the same step as prediction of ICB response, 30 adaptive individualized gene pairs were identified, as the weighted F1-score performance improved with the increase of the number of AIGPS and achieved a balance at approximately 30 pairs except for naive Bayes algorithm (Figure 5A). The discriminative model using random forest, named AIGPS-30, was trained based on 30 adaptive individualized gene pairs. The weighted F1-score, macro F1-score, macro precision, weighted precision, macro recall, weighted recall, and accuracy of AIGPS-30 reached 0.87, 0.88, and 0.86 for 5-class, 3-class, and 2-class skin cancer identification, which improve more than 5%–8% to the benchmark method proposed by Gálvez^12^ (Figure 5B). As the microarray data were only processed by robust multi-array average (RMA) without batch effect removal or further normalization in this experiment, these results demonstrate the generalization ability of AIGPS.

**Table 3:** Datasets partitioning in skin cancer diagnosis

2-class	3-class	5-class	Discovery sets (training set)	Test sets
Healthy			GSE30999 (85), GSE13355 (64), GSE03189 (25)	GSE02503 (6), GSE06710 (13), GSE07553 (4), GSE14905 (21), GSE15605 (16), GSE32407 (10), GSE32924 (8), GSE42677 (10), GSE52471 (13), GSE53462 (5), GSE82105 (6), GSE46517 (16), GSE53223 (18)
Disease	Non-MEL	BCC	GSE07553 (15), GSE42109 (11)	GSE39612 (2), GSE53462 (16)
		SCC	GSE45216 (30), GSE07553 (11)	GSE02503 (5), GSE32628 (15), GSE39612 (4), GSE42677 (10), GSE53462 (5), GSE66359 (8)
		MCC	GSE39612 (30)	GSE36150 (15), GSE50451 (23)
	MEL		GSE07553 (56), GSE15605 (58), GSE03189 (45)	GSE29359 (82), GSE46517 (83), GSE82105 (6)
Total			**430**	**420**

We also evaluated the performance of AIGPS on different processing data to verify the cross-cohort performance of AIGPS, ensuring its effectiveness in different scenarios. Other than applying the RMA preprocessing only, we performed AIGPS on the dataset processed by incorporating batch effect removal along with RMA (RMA + debat; Figures S12A and S12B), and dataset processed by adding normalization to the RMA before batch effect removal (RMA + norm; Figures S12C and S12D). Twenty adaptive individualized gene pairs were identified after RMA + debat or RMA + norm (Figures S12A and S12C) and respective discriminative model using random forest was named AIGPS-20. In this case, the weighted F1-score of AIGPS-20 achieved are 0.95, 0.96, and 0.96 for 5-class, 3-class, and 2-class skin cancer identification with RMA + debat and RMA+norm preprocessing. These results demonstrated that the combination of batch effect removal improves the classification performance. Comparing with the benchmark method using DEGs, AIGPS-20 exceeded in all the metrics except macro recall at 5-class (Figures S12B and S12D). In the dataset with RMA + norm preprocessing, the precisions of AIGPS-20 in 5-class skin cancer classification, namely healthy, BCC, SCC, MCC, and MEL, are 0.93, 1, 0.98, 1, and 0.95, respectively (Figure 5C). The precisions are 0.96, 0.97, and 0.95 for healthy, non-MEL, and MEL (Figure 5D). For distinguishing skin cancer from normal controls, the precision is 0.96 for healthy and 0.97 for disease (Figure 5E).

Besides that, we also compared different coefficients for adaptive difference alteration (Figure S13). We found that a=1 had advantages in binary classification problem when using random forest algorithm, but a=0 was relatively stable on the whole, while a=1or2 had poor performance in multi-classification, so we finally chose a=0. Additionally, due to an ample number of samples, this experiment did not require cross-validation for parameter selection.

### Biological functions of AIGPS in skin cancer diagnosis

For the AIGPS identified for skin cancer classification, we evaluated their feature importance and conducted correlation analysis for the corresponding cancer types. We then built a gene network to visualize gene interactions and performed enrichment analysis to gain insights into their biological functions and pathways.

**Figure 6:**
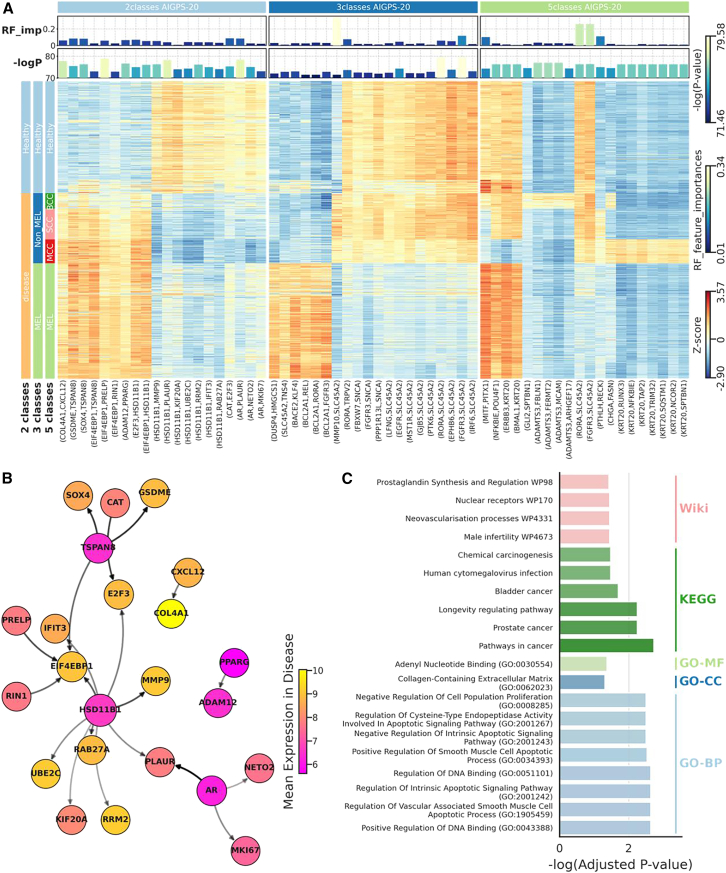
Reversed adaptive gene pairs in AIGPS-20 for skin cancer diagnosis (A) Heatmap displaying the reversed level of gene pair, feature importance of AIGPS-20 in random forest in 5-class, 3-class, and 2-class skin cancer classification. (B) Gene network constructed by 2-class AIGPS-20 in melanoma diagnosis. The arrows on the edges indicate that the mean expression of gene A is higher than that of gene B in melanoma. The transparency of the edge represents the weight of the reversed adaptive gene pairs in the random forest algorithm, and the size of the node represents the degree. (C) Enrichment analysis of the genes involved in 2-class AIGPS-20 in melanoma diagnosis. The top 8 enriched pathways with adjusted *p* value less than 0.05 are displayed in Gene Ontology Cellular Component (GO-CC), Gene Ontology Biological Process (GO-BP), Gene Ontology Molecular Function (GO-MF), KEGG Pathway, Reactome Pathway, and Wiki Pathway. RF-imp, Feature importance in random forest.

We displayed the feature importance, the reversal significance, and the differences of gene pairs in AIGPS-20 identified after RMA+norm (Figure 6A). There are several reversed gene pairs that significantly identify each class, demonstrating that AIGPS-20 can overcome the heterogeneity of samples in cancers. Among these pairs, *MMP10-SLC45A2* has higher feature importances in 3-class to recognize non-MEL heterogeneity, while *RORA-SLC45A2*, *FGFR3-SLC45A2* had higher feature importances in 5-class and 3-class to recognize the heterogeneity of MEL.

We constructed gene network using pairs in AIGPS-20 identified after RMA+norm for 5-class, 3- class, and 2-class (Figures 6B, S14A and S14B), and discovered key genes as skin cancer biomarker, such as *HSD11B1*, *SLC45A2*, *KRT20*, etc. By performing gene enrichment analysis using genes in AIGPS-20, we found that these genes are predominantly associated with cancer and gene regulation (Figures 6C, S14C, and S14D). This suggests that genes identified by AIGPS-20 play important roles in the occurrence, development, and metastasis of cancer.

## Discussion

In this paper, we proposed an algorithm that introduced adaptive differences to screen out alteration of gene pairs in individuals and construct AIGPS for melanoma ICB response prediction and multiclass skin cancer diagnosis. Unlike conventional metrics (e.g., fold change or log2FC) that quantify population-level mean variations, our adaptive difference dynamically adjusts thresholds for individual gene pairs based on their intrinsic standard deviations $a\sqrt{S_{i}^{2}+S_{j}^{2}}$, a design derived from the Welch-Satterthwaite t test. This approach captures intra-individual expression fluctuations and mitigates inter-sample heterogeneity, enhancing robustness against batch effects and technical noise. The AIGPS has better performance in multi-cohort analysis including across single cell and bulk than traditional methods based on absolute gene expression and also make better use of quantitative alteration than rank-based methods. The results demonstrate the superior capability of AIGPS than current methods in predicting ICB response and multi-skin cancer type diagnosis.

Although signatures based on gene pairs can reduce the impact of cross-cohorts and data normalization methods, it loses quantitative information of gene expression. Introducing alteration of difference in gene pair can adopt the quantitative information of genes, but the determination of the difference threshold is tricky, as different gene may have variable fluctuation. The adaptive mechanism not only preserves the comparative advantages of gene pair ranking but also incorporates quantitative expression changes, thereby improving model interpretability and cross-cohort generalizability.

Previous algorithms for classification of transcriptome data often relied on the proper normalization method^57^^,^^58^^,^^59^^,^^60^ and the results of validation of trained models on new cohorts are often unsatisfactory due to the independence and heterogeneity of cohorts. In addition, the complexity of tumor-immune system interactions and the association between intra-tumor heterogeneity^61^^,^^62^^,^^63^^,^^64^^,^^65^ also lead to instability in predictive outcomes. Therefore, some validated biomarkers based on gene expression level are also difficult to use to train a stable prediction model. AIGPS tries to avoid these problems and converts the expression quantity into size relationship comparison and train a robust model. In the skin cancer diagnosis, when using unnormalized data, the AIGPS only lags behind the normalized data by 2–5 percentage points. However, when using DEGs as features, there is a larger gap, with a lag of 12–14 percentage points. Furthermore, AIGPS has the capability to integrate single-cell and bulk data together, similar to previous studies, but with the condition that the data comes from the same tissue.^50^^,^^66^^,^^67^^,^^68^ It is important to note that gene pairs identified using single-cell data may not necessarily be applicable to bulk data. Further exploration of gene pairs is required. For example, from an initial selection of 50 gene pairs in melanoma immune response prediction, we were able to further filter it down to 27 gene pairs, and interestingly, the performance of AIGPS-27 slightly better than that of the original 50 pairs.

In our method, AIGPS primarily relies on intra-sample relative expression relationships of gene pairs combined with an adaptive difference threshold, rather than absolute expression values, which inherently confers robustness against batch effects. Furthermore, in the multi-class skin cancer experiments, we deliberately evaluated AIGPS under varying preprocessing conditions (RMA-only, RMA +debating, and RMA +normalization). The results demonstrate that AIGPS achieves performance comparable to or even superior to conventional methods without batch effect correction, further validating its cross-batch stability (Figures 5A and S12A). For technical replicate assessments, the robustness of AIGPS across heterogeneous experimental conditions and data sources has been extensively verified through test on multiple sequencing platforms (e.g., GPL96, GPL570, and GPL6104; Table S2) and independent cohorts (7 ICB cohorts and 24 skin cancer cohorts).

In order to identify sufficiently significant gene pairs, we typically vary the value of a when processing the discovery set with the AIGPS module. A higher value of a indicates greater differences in the discovered gene pairs. In melanoma immune response prediction, we found that the results with a=2 were better in most cases. However, in skin cancer diagnosis, especially when there are many categories, the value of a is not the larger the better. We attribute the primary cause of this situation to the disparities that exist between the RNA-seq and microarray data types. We also tested L1-regularized logistic regression (LASSO), but its cross-cohort median AUC was significantly lower than RF. This discrepancy likely stems from the low sparsity of gene pair features, where regularization failed to enhance performance (as follow). Nonlinear models (e.g., RF) are better suited to the feature architecture of this study.

Unlike conventional biomarkers—TMB (reflecting genomic instability but lacking dynamic microenvironmental information), CYT (reliant on average immune gene expression and prone to batch effects), or IMPRES (constrained by fixed gene pairs and limited cross-cohort stability)—AIGPS dynamically identifies personalized adaptive gene pairs covering tumor-microenvironment interactions while quantifying expression changes through adaptive differential analysis. Notably, AIGPS retains IMPRES’s capacity to capture key immune checkpoint genes (e.g., *CD86* and *TNFRSF9*) but significantly enhances cross-cohort robustness and biological interpretability. AIGPS also captures melanoma-specific regulatory mechanisms through its selected gene pairs (e.g., *CXCL13-TNFRSF9* and *CDK1-RB1*), such as chemokine signaling pathway, cytokine-cytokine interaction, and retinoblastoma in cancer.

The network built on AIGPS can identify important genes. In melanoma immune response prediction, we identified *MS4A1* as a crucial hub gene, which is consistent with previous studies. *MS4A1* encodes the B cell surface marker *CD20*, is a member of the *MS4A* gene family which has been identified as n potential biomarker for predicting immunotherapy sensitivity in patients with colon cancer (CC) and having potential applications in gene therapy to inhibit CC progression.^69^ Furthermore, *MS4A1* expression level in colorectal carcinoma is positively correlated with patient survival.^70^ And *MS4A1* expression is associated with better prognosis in breast cancer patients and can serve as an independent prognostic factor. In breast cancer patients with high *MS4A1* expression, immune-related pathways are enriched, suggesting an active immune status.^71^ These findings highlight the important roles of *MS4A1* in cancer metastasis, immunotherapy sensitivity, and the regulation of immune cells. In skin cancer diagnosis, *HSD11B1*, *SLC45A2*, and *KRT20* have also been demonstrated to play key roles. *HSD11B1*, an enzyme crucial for regulating glucocorticoid activity in tissues, exhibits expression in various cellular compartments, including myeloid cells, T cells, and melanoma cells. Importantly, high levels of *HSD11B1* expression in melanoma have been linked to poor responses to immune checkpoint inhibitors.^72^ On the other hand, *SLC45A2* encodes a transporter protein that mediates melanin synthesis and shows high expression in uveal melanoma while being present at low levels in normal melanocytes.^73^^,^^74^^,^^75^ Notably, *KRT20* was identified as a key gene associated with lymphatic metastasis and poor prognosis in head and neck squamous cell carcinoma (HNSCC), with overexpression enhancing migration and invasion abilities of cancer cells.^76^^,^^77^ Moreover, understanding these key genes could provide valuable insights into the mechanisms underlying these diseases and potentially guide the development of targeted therapeutics in the future.

AIGPS demonstrates its clinical translational potential. Its design, based on gene-pair relative expression (rank) and adaptive difference, eliminates dependence on absolute expression values and significantly enhances cross-platform generalizability. Although AIGPS requires exhaustive gene-pair screening during feature construction, this computationally intensive process (primarily for *p* value calculation) is performed offline in practice. For clinical deployment, only a fixed set of selected features is needed for model prediction. In our experiments, the RF model required evaluation of merely 27–30 gene pairs, ensuring both computational efficiency and ease of implementation, while maintaining robust anti-batch-effect capability across multiple sequencing platforms. Supported by RNA-seq technologies like targeted RNA-seq, qPCR, and dPCR, AIGPS offers a cost-effective and highly stable molecular diagnostic tool. These platforms are well-suited for its practical implementation: targeted RNA-seq provides high sensitivity and specificity for quantifying all 27 gene pairs, making it scalable and efficient; qPCR offers a cost-effective and rapid option for routine clinical use; and dPCR’s higher sensitivity benefits samples with low or degraded RNA.

In summary, AIGPS extracted from single cell or bulk RNA has demonstrated robust performance on the ICB prediction and diagnosis in cancer. Moreover, the gene networks formed by AIGPS can provide new clues and ideas for the diagnosis and treatment in specific diseases. Future studies can focus on the potential interactions and regulatory modes among these genes, thereby revealing the pathogenesis of diseases or aiding in drug development.

### Limitations of the study

There are also some limitations of this work. AIGPS demonstrates strong batch-effect robustness through relative gene-pair expression and adaptive differences. However, its feature construction requires exhaustive pairwise gene screening, resulting in high computational complexity. Although clinical deployment uses a fixed feature set, the initial computational cost may hinder application in resource-limited settings. Furthermore, gene pairs identified from single-cell data need validation for bulk data applicability, and cross-data-type feature transferability requires systematic evaluation. Regarding parameter optimization, the optimal adaptive coefficient exhibits task-specific characteristics. Significantly different values are required for immunotherapy prediction versus multi-class skin cancer diagnosis. Moreover, observed discrepancies in optimal coefficient values between scRNA-seq and microarray platforms compound implementation complexity. Finally, AIGPS effectiveness is model dependent. It performs well in random forests but suffers significant performance degradation in LASSO models due to feature sparsity. This study did not assess potential sex or gender influences as the datasets did not provide such information. Future studies should validate algorithmic generalizability using broader nonlinear models.

## Resource availability

### Lead contact

Further information and requests for resources and reagents should be directed to and will be fulfilled by the lead contact, Xubin Zheng (xbzheng@gbu.edu.cn).

### Materials availability

This study did not generate new materials.

### Data and code availability

• The melanoma single-cell cohort is available in the GEO (Gene Expression Omnibus) database with series id GSE120575. Riaz cohort is available in the GEO database with series id GSE91061. Hugo cohort is available in the GEO database with series id GSE78220. MGH cohort is available in the GEO database with series ids GSE115821 and GSE168204. Gide cohort is available in the BioProject database with the accession number PRJEB23709. Lee cohort is available in the EGA (European Genome-phenome Archive) with dataset accession number EGAD00001005738. Van cohort is available in dbGaP (The database of Genotypes and Phenotypes) with accession number phs000452.v2.p1. Microarray cohorts are available in the GEO database using their respective series ids. Source data are provided with this paper and can also be obtained from the lead contact.
• All code used in this study is available via https://github.com/ws6tg/AIGPS-main.
• Any additional information required to reanalyze the data reported in this paper is available from the lead contact upon request.

## Acknowledgments

This research was supported by National Natural Science Foundation of China (62176164, 32370711, and 32300554), Shenzhen Medical Research Fund (A2303033), Shenzhen Science and Technology Program (JCYJ20220530152409020, JCYJ20220531101217039, and RKX20220705152810024), and the 10.13039/501100003453Natural Science Foundation of Guangdong Province under grant 2023A1515010992.

## Author contributions

X.Z., Z.D., and L.C. supervised the project and wrote manuscript. X.Z., Z.D., Q.C., and W.H. conceived the idea and designed the experiment. X.Z., Q.C., and W.H. analyzed the data and performed experiments. Y.Z., H.W., and D.W. collected the data. All authors discussed the results and revised the manuscript.

## Declaration of interests

The authors declare no competing interests.

## STAR★Methods

### Key resources table

**Table:** 

REAGENT or RESOURCE	SOURCE	IDENTIFIER
**Deposited data**		

GSE120575	GEO (Gene Expression Omnibus) database	www.ncbi.nlm.nih.gov/geo/query/acc.cgi?acc=GSE120575
Riaz cohort	GEO (Gene Expression Omnibus) database	www.ncbi.nlm.nih.gov/geo/query/acc.cgi?acc=GSE91061
Hugo cohort	GEO (Gene Expression Omnibus) database	www.ncbi.nlm.nih.gov/geo/query/acc.cgi?acc=GSE78220
MGH cohort	GEO (Gene Expression Omnibus) database	www.ncbi.nlm.nih.gov/geo/query/acc.cgi?acc=GSE115821, www.ncbi.nlm.nih.gov/geo/query/acc.cgi?acc=GSE168204
Gide cohort	BioProject database	ngdc.cncb.ac.cn/bioproject/browse/insdc/PRJEB23709
Lee cohort	EGA (European Genome-phenome Archive)	ega-archive.org/datasets/EGAD00001005738
Van cohort	dbGaP (The database of Genotypes and Phenotypes)	www.ncbi.nlm.nih.gov/projects/gap/cgi-bin/study.cgi?study_id=phs000452.v2.p1
GSE02503	GEO (Gene Expression Omnibus) database	www.ncbi.nlm.nih.gov/geo/query/acc.cgi?acc=GSE02503
GSE03189	GEO (Gene Expression Omnibus) database	www.ncbi.nlm.nih.gov/geo/query/acc.cgi?acc=GSE03189
GSE06710	GEO (Gene Expression Omnibus) database	www.ncbi.nlm.nih.gov/geo/query/acc.cgi?acc=GSE06710
GSE07553	GEO (Gene Expression Omnibus) database	www.ncbi.nlm.nih.gov/geo/query/acc.cgi?acc=GSE07553
GSE13355	GEO (Gene Expression Omnibus) database	www.ncbi.nlm.nih.gov/geo/query/acc.cgi?acc=GSE13355
GSE14905	GEO (Gene Expression Omnibus) database	www.ncbi.nlm.nih.gov/geo/query/acc.cgi?acc=GSE14905
GSE15605	GEO (Gene Expression Omnibus) database	www.ncbi.nlm.nih.gov/geo/query/acc.cgi?acc=GSE15605
GSE29359	GEO (Gene Expression Omnibus) database	www.ncbi.nlm.nih.gov/geo/query/acc.cgi?acc=GSE29359
GSE30999	GEO (Gene Expression Omnibus) database	www.ncbi.nlm.nih.gov/geo/query/acc.cgi?acc=GSE30999
GSE32407	GEO (Gene Expression Omnibus) database	www.ncbi.nlm.nih.gov/geo/query/acc.cgi?acc=GSE32407
GSE32628	GEO (Gene Expression Omnibus) database	www.ncbi.nlm.nih.gov/geo/query/acc.cgi?acc=GSE32628
GSE32924	GEO (Gene Expression Omnibus) database	www.ncbi.nlm.nih.gov/geo/query/acc.cgi?acc=GSE32924
GSE36150	GEO (Gene Expression Omnibus) database	www.ncbi.nlm.nih.gov/geo/query/acc.cgi?acc=GSE36150
GSE39612	GEO (Gene Expression Omnibus) database	www.ncbi.nlm.nih.gov/geo/query/acc.cgi?acc=GSE39612
GSE42109	GEO (Gene Expression Omnibus) database	www.ncbi.nlm.nih.gov/geo/query/acc.cgi?acc=GSE42109
GSE42677	GEO (Gene Expression Omnibus) database	www.ncbi.nlm.nih.gov/geo/query/acc.cgi?acc=GSE42677
GSE45216	GEO (Gene Expression Omnibus) database	www.ncbi.nlm.nih.gov/geo/query/acc.cgi?acc=GSE45216
GSE46517	GEO (Gene Expression Omnibus) database	www.ncbi.nlm.nih.gov/geo/query/acc.cgi?acc=GSE46517
GSE50451	GEO (Gene Expression Omnibus) database	www.ncbi.nlm.nih.gov/geo/query/acc.cgi?acc=GSE50451
GSE52471	GEO (Gene Expression Omnibus) database	www.ncbi.nlm.nih.gov/geo/query/acc.cgi?acc=GSE52471
GSE53223	GEO (Gene Expression Omnibus) database	www.ncbi.nlm.nih.gov/geo/query/acc.cgi?acc=GSE53223
GSE53462	GEO (Gene Expression Omnibus) database	www.ncbi.nlm.nih.gov/geo/query/acc.cgi?acc=GSE53462
GSE66359	GEO (Gene Expression Omnibus) database	www.ncbi.nlm.nih.gov/geo/query/acc.cgi?acc=GSE66359
GSE82105	GEO (Gene Expression Omnibus) database	www.ncbi.nlm.nih.gov/geo/query/acc.cgi?acc=GSE82105

**Software and algorithms**		

Scanpy		https://scanpy.readthedocs.io/en/stable/
scikit-learn		https://scikit-learn.org/stable/index.html
Lifelines		https://lifelines.readthedocs.io/en/latest/
Gseapy		https://gseapy.readthedocs.io/en/latest/

### Method details

#### Overall design of the study

In this study, we introduced an adaptively quantitative rank-based method and proposed adaptive individualized gene pair signatures (AIGPS) for multiclass classification of closely related skin cancer types and the prediction of immune responses in melanoma, using integrative transcriptomic data. The workflow of this study encompasses data collection, cohorts partitioning, identification and training of AIGPS, and performance evaluation and comparison (Figure 1A). Transcriptomic profiles, encompassing microarray, RNA sequencing (RNA-seq), and single-cell RNA sequencing (scRNA-seq) data, were collected and divided into distinct datasets: a discovery set, a training set, and test sets. The discovery set, comprising scRNA-seq or bulk RNA data (including microarray and RNA-seq), was utilized for the construction and screening of potential adaptive gene pairs. The training set, consisting of bulk RNA data, was used to construct AIGPS and train the discriminative model. Subsequently, the AIGPS was evaluated and compared across multiple independent test cohorts within the test sets.

The AIGPS module comprises three components: adaptive individualized gene pair construction within individuals, reversed significance calculation and screening, and discriminative model training (Figures 1B–1D). We first took the intersected genes from multiple cohorts and used disease-related genes obtained from NCBI to construct the gene expression matrix as input (Figure 1B). To build adaptive individualized gene pairs, we performed exhausted comparison between every two genes within each individual and introduced an adaptive difference derived from the Welch-Satterthwaite's T-test to capture the difference alteration between genes. For example, the expression of gene i, denoted as $G_{i}$, was considered greater than gene j , denoted as $G_{j}$, only if ${\;G}_{i,k}-G_{j,k}>a\sqrt{S_{i}^{2}+S_{j}^{2}}$, where $S_{i}$, $S_{j}$ is the standard deviation of gene i, j and a is the coefficient (Figure 1C). $a\sqrt{S_{i}^{2}+S_{j}^{2}}$ can describe the quantitative alteration adaptive to different genes. After that, we performed cross-population analysis to obtain those gene pairs that were significantly reversed among different cancer types or in ICB response as potential signatures. Fisher's exact test or Pearson’s chi-squared test for multiclass were applied to calculate the significance level (P value) for initial screening (Figure 1D). After training and comparing different machine learning methods on potential signatures, random forest was used to further extract the gene pair signatures based on the feature importance of reversed gene pairs and trained as discriminative model. Through these processes, AIGPS can have better generalization capabilities and explainability.

#### Cohorts collected in immunotherapy response prediction

In our study, we collected data from a single-cell transcriptomic profile of melanoma GSE120575,^52^ consisting of 5928 pre-treatment cells, and six bulk RNA-seq profile of melanoma, namely Riaz,^78^ Van,^26^ Hugo,^79^ Gide,^80^ Lee^81^ and MGH,^35^ comprising 252 samples (Table 1). To enhance clarity in data partitioning, we have added Figure S1 to explicitly illustrate the specific usage of datasets throughout the workflow.

In the GSE120575 cohort, there were 48 biopsies from 32 patients treated with anti-PD1 (35 biopsies), anti-CTLA4+PD1 (11 biopsies) and anti-CTLA4 (2 biopsies), including 19 pre-treatment biopsies and 29 post-treatment biopsies. According to response evaluation criteria in solid tumors (RECIST) criteri^82^: samples with complete response (CR), partial response (PR), or stable disease (SD) with progression-free survival (PFS) longer than 180 days are classified as responders and samples with progressive disease (PD) or SD with PFS shorter than 180 days were classified as non-responders, the 48 biopsies can be divided into 17 responders and 31 non-responders. In this study, we only used pre-treatment biopsies, which contained 5928 pre-treatment cells with 2725 response cells and 3203 non-response cells.

In the Riaz cohort, there were 108 biopsies from 68 patients treated with anti-PD1 (59 biopsies), and anti-PD1+prior anti-CTLA4 (49 biopsies). After filtering out samples without bulk RNA-seq data or RECIST, 49 pre-treatment biopsies with 18 responders and 31 non-responders were obtained. In the Van cohort, there were 42 biopsies from 40 patients treated with anti-CTLA. After filtering out samples without bulk RNA-seq data or RECIST, 41 pre-treatment biopsies with 12 responders and 29 non-responders were obtained. In the Hugo cohort, there were 39 biopsies from 38 patients treated with anti-PD1. After filtering out samples without bulk RNA-seq data or RECIST, 27 pre-treatment biopsies with 15 responders and 12 non-responders were obtained. In the Lee cohort, there were 94 biopsies from 55 patients treated with anti-PD1. After filtering out samples without bulk RNA-seq data or RECIST, we get 44 pre-treatment biopsies with 22 responders and 22 non-responders were obtained. In the Gide cohort, there were 121 biopsies from 54 patients treated with anti-PD1 (63 biopsies) and anti-CTLA4+PD1 (58 biopsies). After filtering out samples without bulk RNA-seq data or RECIST, 72 pre-treatment biopsies with 45 responders and 27 non-responders were obtained. In the MGH cohort, there were 50 biopsies from 81 patients treated with anti-PD1 (32 biopsies), anti-PDL1 (8 biopsies) and anti-CTLA4+PD1 (10 biopsies). After filtering out samples without bulk RNA-seq data or RECIST, 19 pre-treatment biopsies with 6 responders and 13 non-responders were obtained (Table 1). We took intersection of genes among bulk RNA cohorts profiling 16,128 genes, the single-cell cohort GSE120575 profiling 12364 genes, and 2,613 melanoma-related genes from NCBI, and obtained 1425 for further analysis.

In the melanoma immunotherapy response prediction, the scRNA-seq cohort GSE120575 is used as discovery set, the RNA-seq cohorts Riaz and Van are used as training set, and the rest RNA-seq cohorts as the test set. For both scRNA-seq data and RNA-seq data, all of the gene expression matrices were normalized to the transcripts per million (TPM).

#### Cohorts collected in skin cancer diagnosis

We collected transcriptomic profiles of 330 melanoma (MEL), 44 basal cell carcinoma (BCC), 68 Merkel cell carcinoma (MCC), 88 squamous cell carcinoma (SCC) and 320 healthy samples from 24 microarray cohorts which included 850 samples in total (Table 3). The healthy samples contain healthy skin and healthy nevus. The MEL samples contain primary melanoma and metastatic melanoma. The gene chips used in the DNA microarray include Affymetrix and Illumina. Each cohort can be downloaded by searching for its corresponding Series number query listed in Table 2.

In the melanoma diagnosis, the discovery sets and training sets are shared. The microarray cohort assignment is shown in the Table 3, where the discovery set has 430 samples and the test set has 420 samples. We will also make sure that the training set and the test set have roughly the same number of samples to ensure better classification results.

For microarray data, we used the R packages to acquire the gene expression matrices from CEL files, such as affy and oligo for Affymetrix gene chip and lumi for Illumina gene chip, and then annotate genes according to their chip annotation files. The obtained gene expression matrix is subject to RMA normalization and log2 transformation. Subsequent processing included batch effect removal and normalization to a uniform distribution space.

#### Adaptive individualized gene pair signature module

The adaptive individualized gene pair signature (AIGPS) module aims at transforming the gene expression features into adaptive gene pairs, balancing between the generalizability of rank-based gene pair and the alteration in value of absolute gene expression. To refine the selection, we focused on highly variable expressed and disease-associated genes and also took intersection among all the cohorts. The disease-associated genes were retrieved from the NCBI Gene database.

The gene expression matrix can be modeled as a two-order tensor of $G\in R^{m\times n}$, where m and n are the gene number and sample size. The entry ${\;G}_{i,k}$ represents the value of gene i∈{1,⋯,m} in the k∈{1,⋯,n} sample in gene expression matrix. Then we constructed a gene pairs matrix $P\in R^{C_{m}^{2}\times n}$,where $C_{m}^{2}$ and n represents the pair number and sample size. The entry ${\;P}_{l,k}$ represents the value of pair $l\in \left\{1,\cdots ,C_{m}^{2}\right\}$ in the k∈{1,⋯,n} sample in gene pair matrix. Assumed that ${\;G}_{i,k}$ and $G_{j,k}$ are normally distributed as ${\;G}_{i,k}∼N\left(\mu_{i},{\sigma_{i}}^{2}\right),G_{j,k}∼N\left(\mu_{j},{\sigma_{j}}^{2}\right)$, where $\mu_{i},\sigma_{i},\mu_{j},\sigma_{j}$ are unknown parameters. Therefore, we obtained $\bar{G_{i}}∼N\left(\mu_{i},\frac{{\sigma_{i}}^{2}}{n}\right),\bar{G_{j}}∼N\left(\mu_{j},\frac{{\sigma_{j}}^{2}}{n}\right)$, where $\bar{G_{i}}=\frac{1}{n}\sum_{k=1}^{n}G_{i,k}$. According to Welch–Satterthwaite equation,^83^ we deduced

(Equation 1) $$\begin{array}{c} T=\frac{\bar{G_{i}}-\bar{G_{j}}}{\sqrt{\frac{S_{i}^{2}}{n}+\frac{S_{j}^{2}}{n}}}∼t\left(\nu \right) \end{array}$$

where $S_{i}^{2}$ represents the sample variance of the amount of gene i expressed in all samples and can be deduced as

(Equation 2) $$\begin{array}{c} S_{i}^{2}=\frac{1}{\left(n-1\right)}\sum_{k=1}^{n}\;{\left({\;G}_{i,k}-\bar{G_{i}}\right)}^{2}, \end{array}$$

and ν was the degree of freedom calculated via

(Equation 3) $$\begin{array}{c} \nu =\frac{\left(n-1\right){\left(S_{i}^{2}+S_{j}^{2}\right)}^{2}}{S_{i}^{4}+S_{j}^{4}} \end{array}$$

Regarding statistical assumptions for single-cell RNA-seq data, the challenges of distributional assumptions in scRNA-seq modeling have been systematically discussed by Bacher et al.,^84^ notably highlighting that while gene expression data often deviate from normality, approximate methods like the t-test remain robust with adequate sample sizes. This perspective indirectly supports the methodological soundness of statistical threshold selection in AIGPS. Since $\frac{{\;G}_{i,k}}{\sqrt{n}\;}$ and $\bar{G_{i}}$ are identically distributed, by the definition of Student’s t-distribution, we can replace $\frac{{\;G}_{i,k}-G_{j,k}}{\sqrt{n}\;}$ with $\bar{G_{i}}-\bar{G_{j}}$ in formula (1) and obtain

(Equation 4) $$\begin{array}{c} T=\frac{{\;G}_{i,k}-G_{j,k}}{\sqrt{S_{i}^{2}+S_{j}^{2}}}∼t\left(\nu \right) \end{array}$$

Then,

(Equation 5) $$\begin{array}{c} T=\left({\;G}_{i,k}-G_{j,k}\right)∼t\left(\nu \right)\sqrt{S_{i}^{2}+S_{j}^{2}} \end{array}$$

Let a be some quantile of t(ν), then ${\;G}_{i,k}$ was considered to be significantly greater than $G_{j,k}$ if ${\;G}_{i,k}-G_{j,k}>a\sqrt{S_{i}^{2}+S_{j}^{2}}$. Therefore, the value of ${\;P}_{l,k}$ in gene pairs matrix can be defined as

(Equation 6) $$\begin{array}{c} P_{l,k}=\left\{ \begin{array}{c} 1,if{\;G}_{i,k}-G_{j,k}>a\sqrt{S_{i}^{2}+S_{j}^{2}}\\ -1,if\;G_{j,k}-{\;G}_{i,k}>a\sqrt{S_{i}^{2}+S_{j}^{2}}\\ 0,otherwise \end{array}\right.\text{,} \end{array}$$

where $l=\left[\sum_{b=1}^{i-1}\left(m-b\right)\right]+j-i$.

For pair l, $P_{l}=\left(P_{l,0},\cdots ,P_{l,n}\right)$ represents the values of pair l in gene pair matrix in n samples. Since n samples can be classified into z classes, ${\;C}_{k}\in \{1,\cdots ,z\}$ represented the category of k sample. We counted the number of 1 and -1 in each category in $P_{l}$ and obtained a contingency table $T\in R^{2\times z}$, $T_{1,j}$ represents the number of occurrences in all $P_{l}$ where $P_{l,k}=1$ and $j{=C}_{k}$, $T_{2,j}$ represents the number of occurrences in all $P_{l}$ where $P_{l,k}=-1$ and $j{=C}_{k}$, which were shown in the table below:

Class 1 ⋯ Class z

$$P_{l,k}=1\;{T_{1,1}=\text{Num}}_{P_{l,k}=1,{\;C}_{k}=1}\cdots {T_{1,z}=\text{Num}}_{P_{l,k}=1,{\;C}_{k}=z}$$

$$P_{l,k}=-1\;T_{2,1}={\text{Num}}_{P_{l,k}=-1,{\;C}_{k}=1}\cdots T_{2,z}={\text{Num}}_{P_{l,k}=-1,{\;C}_{k}=z}$$

Then T is used to calculate the *p*-value of pair l. For ICB response prediction where z was 2, we calculated the *p*-value of each pair using the Fisher’s exact test:

(Equation 7) $$\begin{array}{c} p=\frac{\left(\begin{array}{c} T_{1,1}+T_{2,1}\\ T_{1,1} \end{array}\right)\left(\begin{array}{c} T_{1,2}+T_{2,2}\\ T_{1,2} \end{array}\right)}{\left(\begin{array}{c} T_{1,1}+{\;T}_{2,1}+{\;T}_{1,2}+T_{2,2}\\ T_{1,1}+T_{1,2} \end{array}\right)}=\frac{\left(T_{1,1}+T_{2,1}\right)!\left(T_{1,2}+T_{2,2}\right)!\left(T_{1,1}+T_{1,2}\right)!\left(T_{2,1}+T_{2,2}\right)!}{T_{1,1}!T_{2,1}!T_{1,2}!T_{2,2}!\left(T_{1,1}+{\;T}_{2,1}+{\;T}_{1,2}+T_{2,2}\right)!} \end{array}$$

For multiple skin cancer types classification tasks, we calculated the *p*-value of each pair using the Pearson’s chi-squared test:

(Equation 8) $$\begin{array}{c} \chi_{Pearson}^{2}=N\left(\sum_{i=1}^{2}\;\sum_{j=1}^{z}\;\frac{T_{i,j}^{2}}{\sum_{c=i}^{z}\;T_{i,c}\sum_{r=1}^{2}\;T_{r,j}}-1\right)∼\chi^{2}\left(z-1\right) \end{array}$$

where N represents the sum of the quantities satisfying $P_{l,k}=1\;or\;P_{l,k}=-1$ in n samples. The *p*-value is the right-tail probability when the test statistic is $\chi_{Pearson}^{2}$.

We utilized the identical method to compute P-value for all pairs and arranged them from smallest to largest by P-value. After that, we compared the performance of different number of pairs with different machine learning models and selected top x significant adaptive gene pairs as potential signature.

We trained the machine learning models based on the top x significant pairs and applied random forest after comparison among seven models including random forest classifier (RF), k-nearest neighbors (KNN), logistics regression (LR), support vector machine (SVM), multi-layer perception (MLP), extreme gradient boosting (XGB) and Naive Bayes (NB). We used three-fold cross-validation to select the optimal parameter combination in building the prediction model. However, for excessive sample size in skin cancer diagnosis, default parameters were used for all models without cross-validation. Finally, we identified the adaptive individualized gene pair signature using random forest with feature importance larger than 0 and build the final ICB response prediction and classification model using random forest.

### Evaluation

To evaluation the performance of our model in predicting ICB response, we used AUC (Area Under the Curve) as the metric. In skin cancer classification, we used accuracy (acc), precision, recall and F1-score as the metric to evaluation the performance of multiclass classification. $P_{m}$, $R_{m}$ and ${F1}_{m}$ represented macro precision, macro recall and macro F1-score, while $P_{w}$, $R_{w}$ and ${F1}_{w}$ represented weighted precision, weighted recall and weighted F1-score. The formulas were as follows:

(Equation 9) $$\begin{array}{c} P_{m}=\frac{\sum_{i=1}^{n}P_{i}}{n} \end{array}$$

(Equation 10) $$\begin{array}{c} R_{m}=\frac{\sum_{i=1}^{n}R_{i}}{n} \end{array}$$

(Equation 11) $$\begin{array}{c} {F1}_{m}=\frac{2\times P_{m}\times R_{m}}{P_{m}+R_{m}} \end{array}$$

(Equation 12) $$\begin{array}{c} P_{w}=\sum_{i=1}^{n}{W_{i}\times P}_{i} \end{array}$$

(Equation 13) $$\begin{array}{c} R_{w}=\sum_{i=1}^{n}{W_{i}\times R}_{i} \end{array}$$

(Equation 14) $$\begin{array}{c} {F1}_{w}=\frac{2\times P_{w}\times R_{w}}{P_{w}+R_{w}} \end{array}$$

where n represents the number of categories, $W_{i}$ represents the proportion of the number of class i in the number of all samples, and $P_{i}$ and $R_{i}$ represents the precision and recall of class i in the case of binary classification:

(Equation 15) $$\begin{array}{c} P=\frac{TP}{TP+FP} \end{array}$$

(Equation 16) $$\begin{array}{c} R=\frac{TP}{TP+FN} \end{array}$$

where FP (False Positive) refers to the number of samples that are incorrectly predicted as positive by the model, TN (True Negative) represents the number of samples that are correctly predicted as negative by the model, FN (False Negative) indicates the number of samples that are incorrectly predicted as negative by the model.

#### Survival analysis

For each sample, AIGPS using random forest provided a probability of ICB response. We calculated the odds ratio for each sample in the cohort and determined the mean of odds ratios. Samples with odds ratios greater than the mean value are classified as the low-risk group, while samples with odds ratios lower than the mean value are classified as the high-risk group. We conducted Kaplan-Meier survival analysis to assess the overall survival and progression-free survival of the patient cohort. The survival outcomes between the low-risk and high-risk groups were compared using a two-sided log-rank test. To quantify the risk associated with each group, we calculated the hazard ratio along with its corresponding confidence interval using univariate Cox proportional hazards models.

#### Network construction

We constructed an AIGPS association network using genes as nodes and connecting gene pairs as edges. The size of each node reflects its degree, indicating its connectivity within the network. The depth of the edge color represents the feature importance of the gene pair. Node color represents the average gene expression. In binary classification, directed edges indicate greater-than relationships in specific classes.

#### Functional analysis

Gene Set Enrichment Analysis (GSEA) was conducted using the GSEApy Python software package. The analysis involved several databases, including GO Molecular Function 2023, GO Biological Process 2023, GO Cellular Component 2023, Reactome 2022, KEGG 2021 Human and WikiPathway 2021 Human. To determine enriched gene sets, the results were sorted based on the adjusted P-value for each database. Specifically, only the top entries with an adjusted P-value less than 0.05 were considered significant.

### Quantification and statistical analysis

All statistical analyses were conducted using Python. The AIGPS algorithm implemented the Welch-Satterthwaite t-test to determine adaptive thresholds for gene expression differences, with coefficient a optimized to 2.0 for immune checkpoint blockade (ICB) response prediction and 0 for multi-class skin cancer classification.

Significant gene pairs were identified using Fisher's exact test for ICB response analysis and Pearson's chi-squared test for skin cancer classification. Predictive modeling was performed using Random Forest algorithm (scikit-learn v1.3.1) with three-fold cross-validation to ensure robust performance estimation. Model evaluation metrics included area under the curve (AUC) for ICB response prediction and both macro and weighted F1-scores for skin cancer classification.

For survival analysis, we employed Kaplan-Meier curves with log-rank tests for significance evaluation, complemented by Cox proportional hazards models to estimate hazard ratios. Functional enrichment analysis was conducted using GSEApy to examine pathway associations from multiple databases including GO, Reactome, KEGG and WikiPathway, with statistical significance threshold set at adjusted p-value < 0.05.

The study analyzed 5,928 single cells for discovery purposes and 252 bulk RNA-seq samples for training and testing. All analytical procedures incorporated fixed random seeds to ensure reproducibility of results. Complete statistical parameters and methodological details are documented in the corresponding figure legends and method details section.
