# Supplementary material for: Adaptive individualized gene pair signatures distinguishing melanoma and predicting response to immune checkpoint blockade
Source: iScience. 2025 Aug 8;28(9):113329. doi: 10.1016/j.isci.2025.113329 (PMC12410410; doi:10.1016/j.isci.2025.113329)
Supplement: Document S1. Figures S1–S14 and Tables S1–S3 [file mmc1.pdf]

iScience, Volume *28*

Supplemental information

Adaptive individualized gene pair signatures distinguishing melanoma and predicting response to immune checkpoint blockade

Zhihua Du, Qiyi Chen, Weiliang Huang, Yijun Zhou, Huaijin Wen, Di Wang, Yinghua Chen, Lixin Cheng, and Xubin Zheng

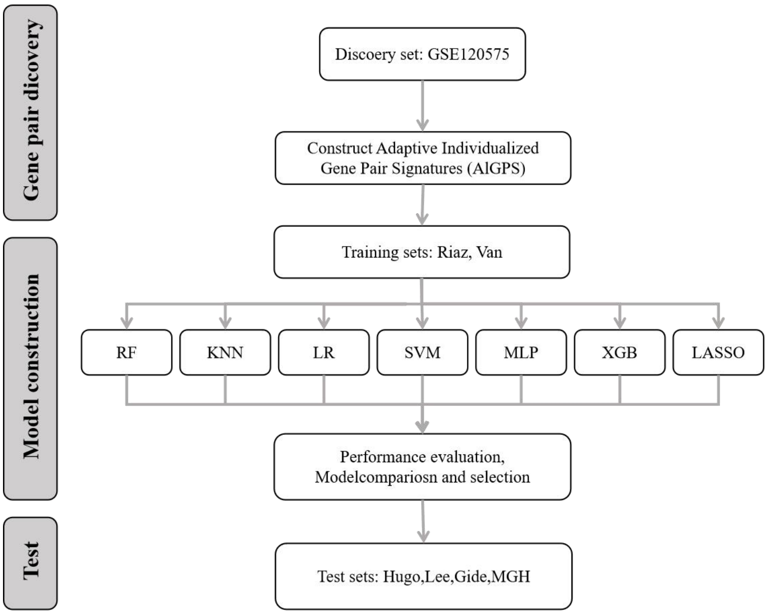

Figure S1. AIGPS-based workflow framework for predicting immune therapy response. Related to STAR Methods.

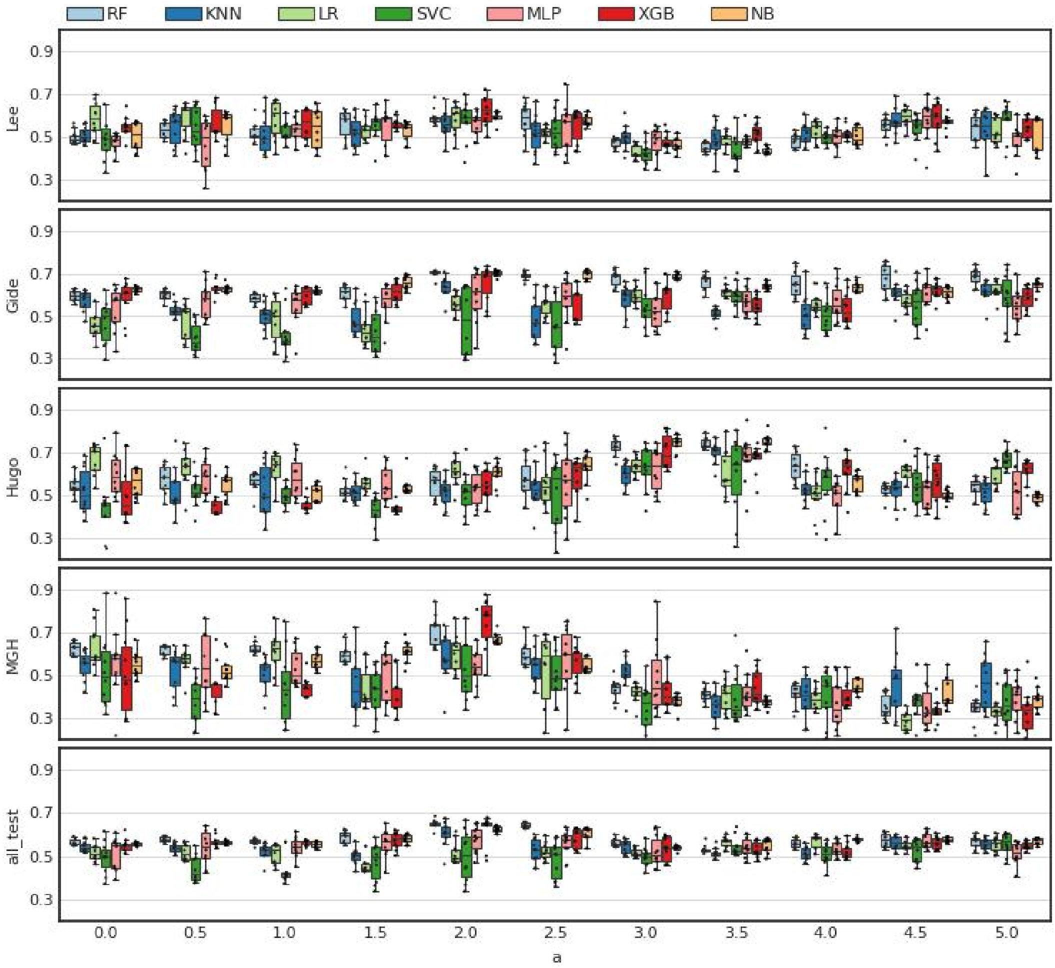

Figure S2. Performance of different adaptive coefficients combined with machine

learning methods in the independent validation cohort. Related to Figure 3.

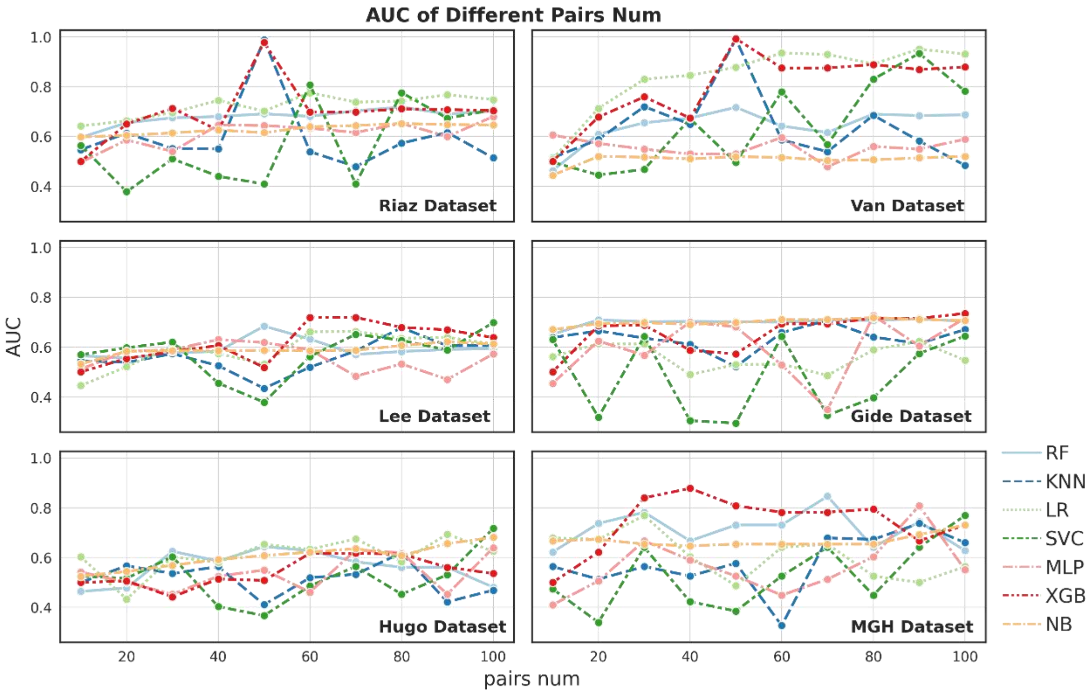

Figure S3. The AUC of various machine learning methods with different numbers of reversed adaptive gene pairs on training cohorts and validation cohorts. Related to STAR Methods.

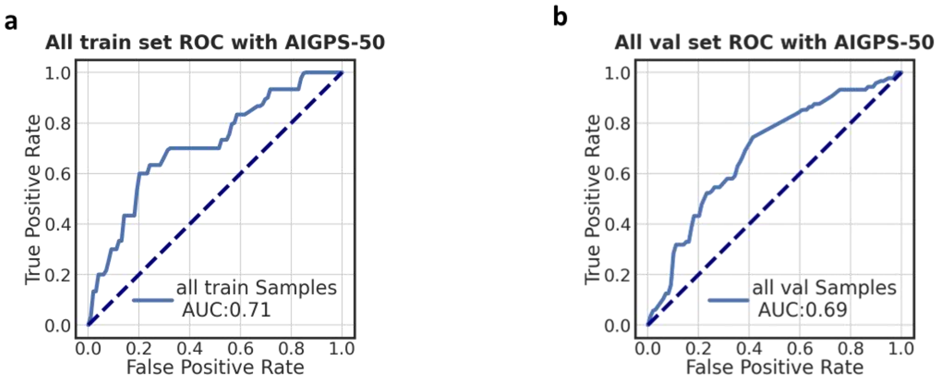

Figure S4. Performance of AIGPS-50. **a** Performance of AIGPS-50 in overall training sets. **b** Performance of AIGPS-50 in validation sets. Related to Figure 3.

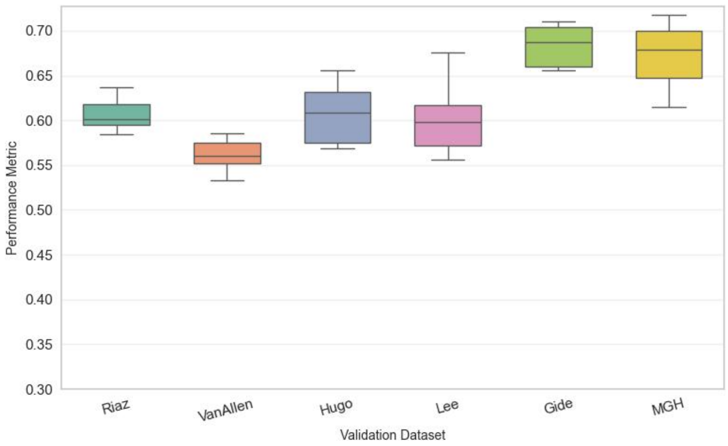

Figure S5. Leave-one-out cross-dataset robustness assessment of model performance in the independent validation cohort. Related to STAR Methods.

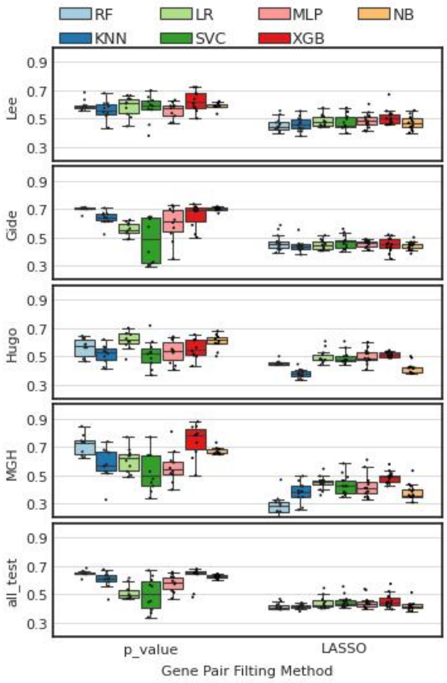

Figure S6. Performance of different feature selection methods combined with machine learning methods in the independent validation cohort. Related to STAR Methods.

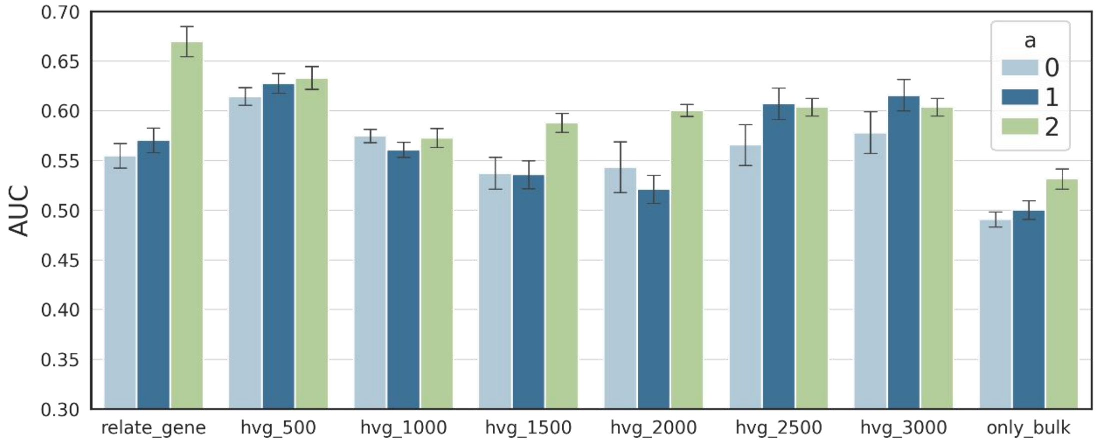

Figure S7. The performance of AIGPS-50 under different adaptive coefficients. Related to STAR Methods.

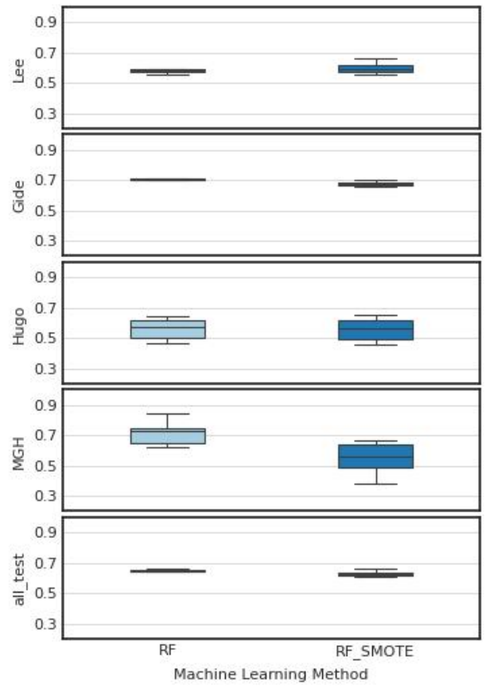

Figure S8. Performance comparison of SMOTE-balanced Random Forest versus other machine learning methods in the independent validation cohort. Related to STAR Methods.

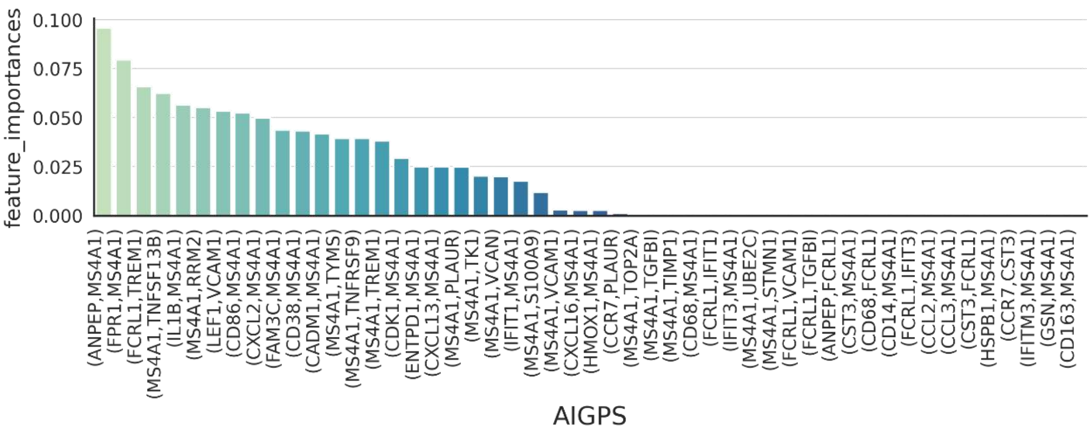

Figure S9. Feature importance of 50 reversed adaptive gene pairs in AIGPS-50 in predicting ICB response. Related to STAR Methods.

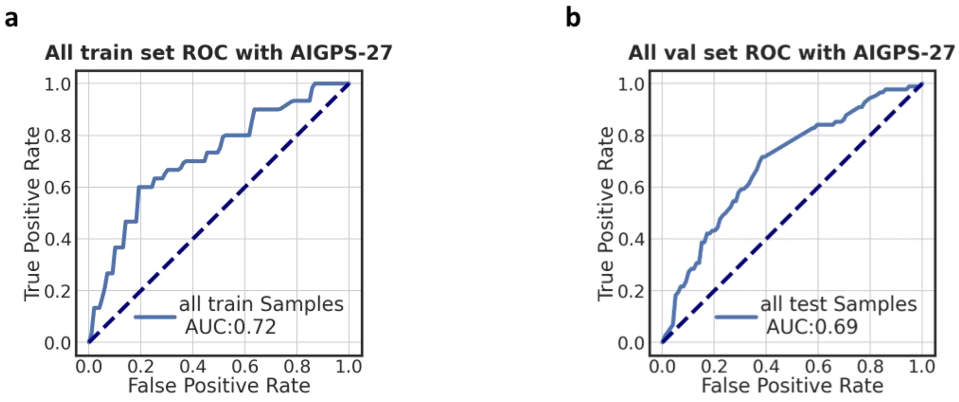

Figure S10. Performance of AIGPS-27. **a** Performance of AIGPS-27 in overall training sets. **b** Performance of AIGPS-27 in validation training sets. Related to STAR Methods.

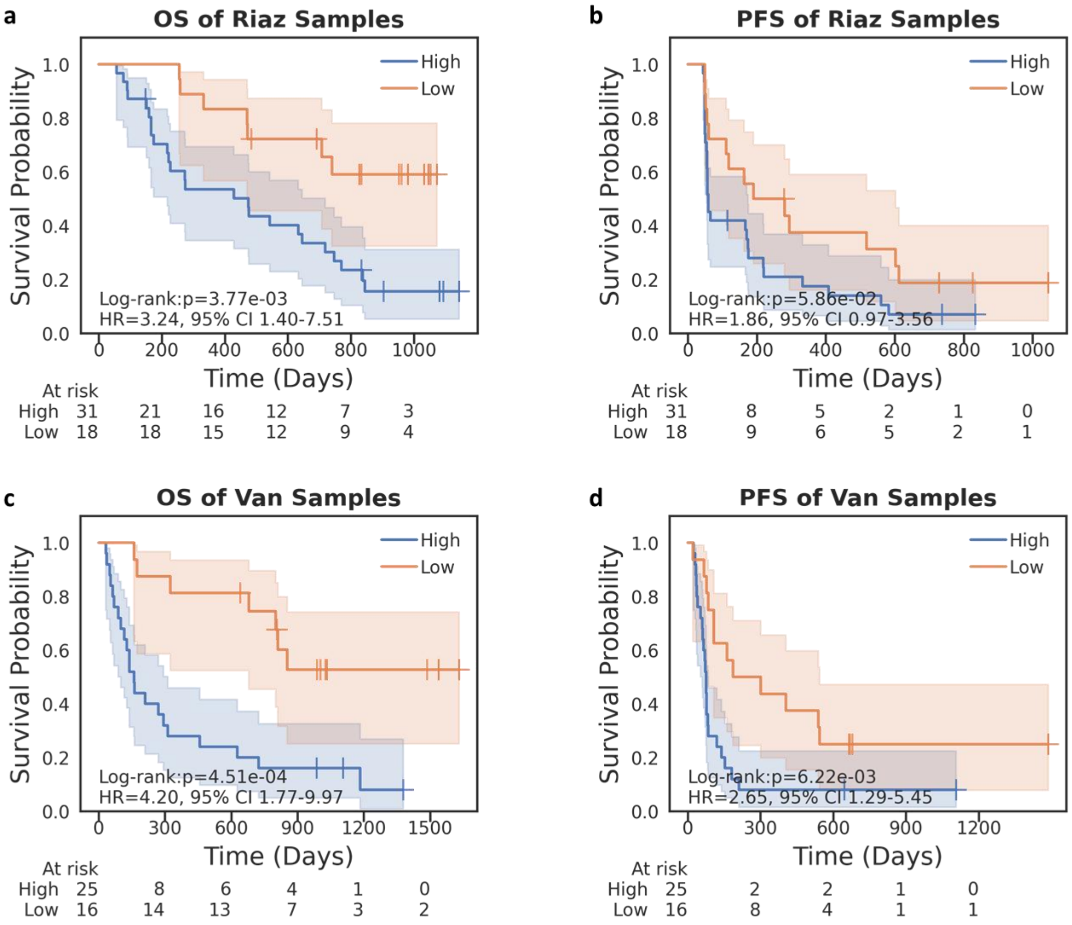

Figure S11. Overall survival (OS) and progression-free survival (PFS) Kaplan-Meier survival curve of samples in Riaz cohort (**a** and **b**) and Van cohort (**c** and **d**), where patients were divided into two groups based on the mean of samples' odd ratio. Statistical analysis is conducted using a two-sided log-rank test, and the Hazard ratio and 95% confidence interval are provided. Related to Figure 4.

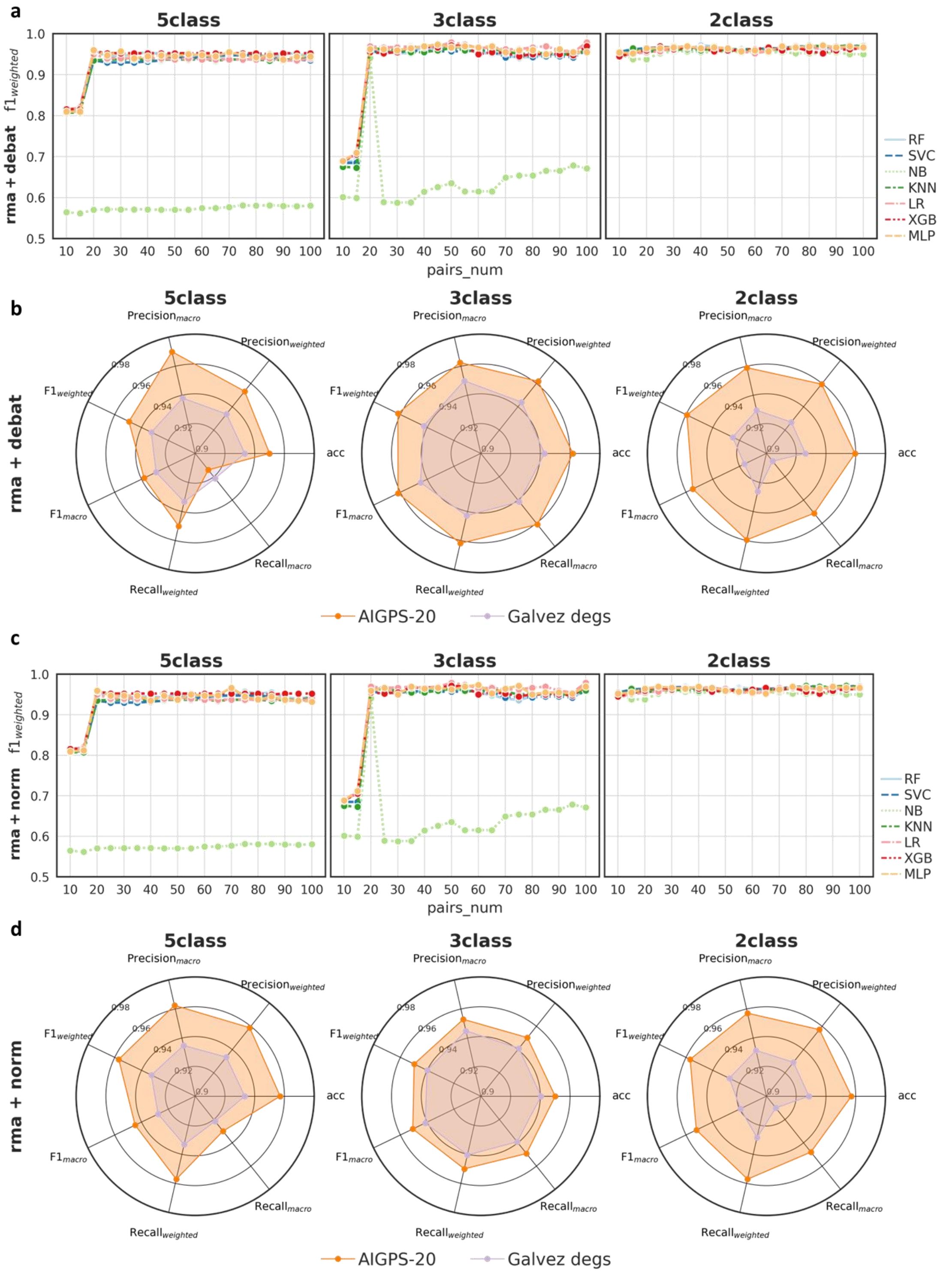

Figure S12. Performance of AIGPS-20 in skin cancer diagnosis **a** The weighted F1-score of various machine learning methods with different numbers of reversed adaptive gene pairs on independent validation cohorts preprocessed with RMA and debat batch removal. **b** Performance of AIGPS comparing to Galvez's differentially expression genes in two-class classification (healthy and disease), three-class classification (healthy, Non-MEL and MEL) and five-class classification (healthy, BCC, SCC, MCC and MEL) on independent validation cohorts preprocessed with RMA and

debat batch removal. **c** The weighted F1-score of various machine learning methods with different numbers of reversed adaptive gene pairs on independent validation cohorts preprocessed with RMA, debat batch removal and further normalization. **d** Performance of AIGPS comparing to Galvez's differentially expression genes in two-class classification (healthy and disease), three-class classification (healthy, Non-MEL and MEL) and five-class classification (healthy, BCC, SCC, MCC and MEL) on independent validation cohorts preprocessed with RMA, debat batch removal and further normalization. Related to Figure 5.

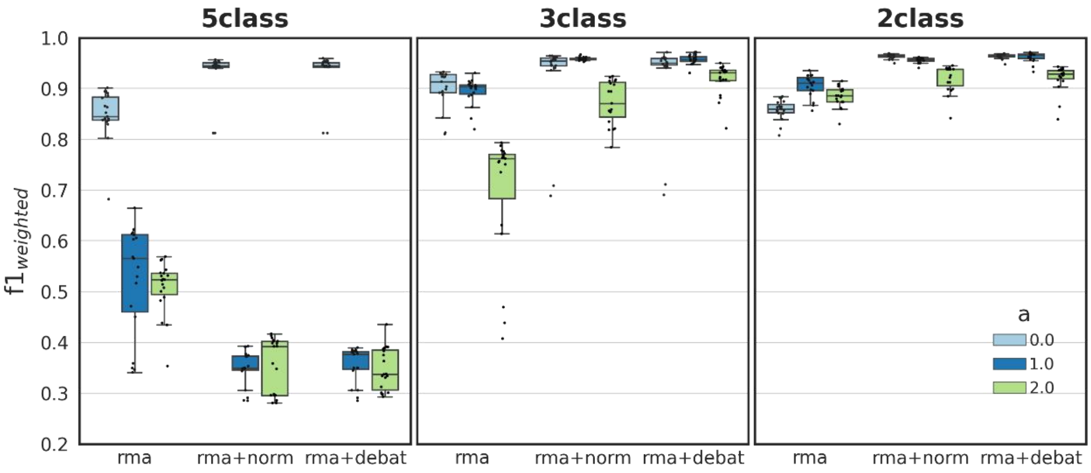

Figure S13. Boxplot displaying the weighted F1-score of different adaptive coefficients and machine learning methods in two-class classification (healthy and disease), three-class classification (healthy, Non-MEL and MEL) and five-class classification (healthy, BCC, SCC, MCC and MEL) on independent validation cohorts. Related to Figure 5.

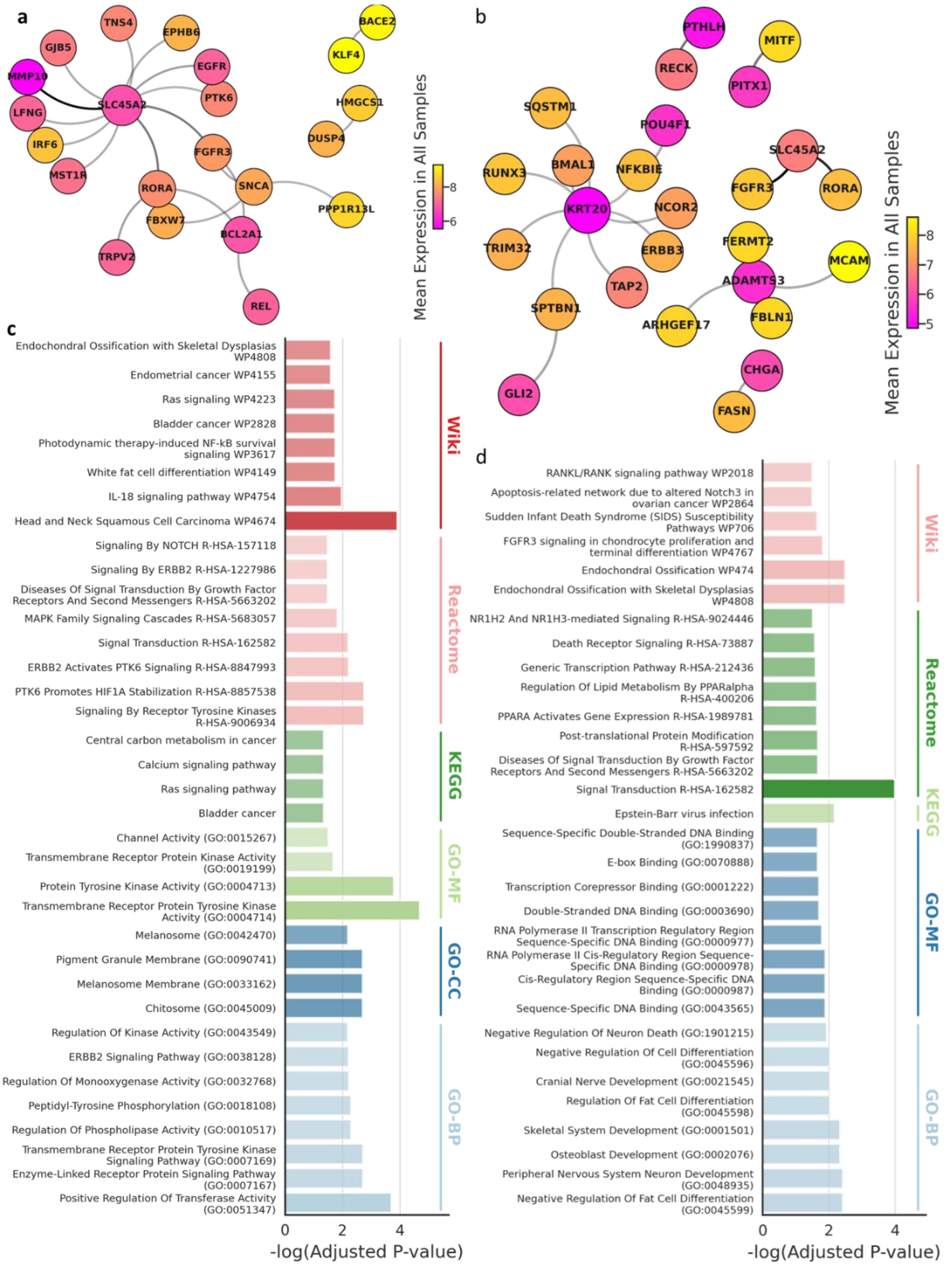

Figure S14. AIGPS analysis in melanoma diagnosis. a Gene network constructed by 3-class AIGPS-20 in melanoma diagnosis. **b** The Gene network constructed by 5-class AIGPS-20 in melanoma diagnosis. The transparency of the edge represents the weight of the Pairs in the random forest algorithm, and the size of the node represents the degree of the node. **c** Enrichment analysis of the genes involved in 3-class AIGPS-20 in melanoma diagnosis. **d** Enrichment analysis of the genes involved in 5-class AIGPS-20 in melanoma diagnosis. The top eight enrichment results with an adjusted p-value less than 0.05 are displayed in Gene Ontology Cellular Component (GO-CC), Gene Ontology Biological Process (GO-BP), Gene Ontology Molecular Function (GO-MF), KEGG Pathway, Reactome Pathway, and

Wiki Pathway. Related to Figure 6.

Table S1 Existing transcriptome-based predictive signatures

Signature name	Signature	Original study
IMPRES	PDCD1-TNFSF4 CD27-PDCD1 CTLA4-TNFSF4 CD40-CD28 CD86-TNFSF4 CD28-CD86 CD80-TNFRSF9 CD274-VSIR CD86-HAVCR2 CD40-PDCD1 CD86-CD200 CD40-CD80 CD28-CD276 CD40-CD274 TNFRSF14-CD86	Auslander, N. et al. Robust prediction of response to immune checkpoint blockade therapy in metastatic melanoma. Nat Med 24, 1545–1549 (2018).
PASS_PRE	COMPLEMENT_CASCADE REGULATION_OF_INSULIN_LIKE_GROWTH_FACTOR_IGF_TRANSPORT_AND_UPTAKE_BY_INSULIN_LIKE_GROWTH_FACTOR_BINDING_PROTEINS_IGFBPS FORMATION_OF_FIBRIN_CLOT_CLOTTING_CASCADE INTRINSIC_PATHWAY_OF_FIBRIN_CLOT_FORMATION PHASE_I_FUNCTIONALIZATION_OF_COMPOUNDS RESPONSE_TO_ELEVATED_PLATELET_CYTOSOLIC_CA2 BILE_ACID_AND_BILE_SALT_METABOLISM PLASMA_LIPOPROTEIN_REMODELING BIOLOGICAL_OXIDATIONS BINDING_AND_UPTAKE_OF_LIGANDS_BY_SCAVENGER_RECEPTORS PLASMA_LIPOPROTEIN_ASSEMBLY INTERLEUKIN_2_FAMILY_SIGNALING SYNTHESIS_OF_BILE_ACIDS_AND_BILE_SALTS RA_BIOSYNTHESIS_PATHWAY GLUCURONIDATION	Du, K. et al. Pathway signatures derived from on-treatment tumor specimens predict response to anti-PD1 blockade in metastatic melanoma. Nat Commun 12, 6023 (2021).
IFN_γ	IFNG STAT1 CXCL9 CXCL10 IDO1 HLA-DRA	Ayers, M. et al. IFN-γ–related mRNA profile predicts clinical response to PD-1 blockade. J Clin Invest 127, 2930–2940 (2017).
T_cell_inflamed	TIGIT CD27 CD8A PDCD1LG2 LAG3 CD274 CXCR6 CMKLR1 NKG7 CCL5 PSMB10 IDO1 CXCL9 HLA-DQA1 CD276 STAT1 HLA-DRB1 HLA-E	Ayers, M. et al. IFN-γ–related mRNA profile predicts clinical response to PD-1 blockade. J Clin Invest 127, 2930–2940 (2017).
MHC-I	TAP1 B2M HLA-A HLA-B HLA-C TAP2	Liu, D. et al. Integrative molecular and clinical modeling of clinical outcomes to PD1 blockade in patients with metastatic melanoma. Nat Med 25, 1916–1927 (2019).
MHC-II	HLA-DMA HLA-DMB HLA-DOB HLA-DO	Liu, D. et al. Integrative molecular and clinical modeling of clinical outcomes to PD1 blockade in patients with metastatic melanoma. Nat Med 25, 1916–1927 (2019).

Immune Score	GZMA GZMB PRF1 GNLY HLA-A HLA-B HLA-C HLA-E HLA-F HLA-G HLA-H HLA-DMA HLA-DMB HLA-DOA HLA-DOB HLA-DPA1 HLA-DPB1 HLA-DQA1 HLA-DQA2 HLA-DQB1 HLA-DRA HLA-DRB1 IFNG IFNGR1 IFNGR2 IRF1 STAT1 PSMB9 CCR5 CCL3 CCL4 CCL5 CXCL9 CXCL10 CXCL11 ICAM1 ICAM2 ICAM3 ICAM4 ICAM5 VCAM1	Roh, W. et al. Integrated molecular analysis of tumor biopsies on sequential CTLA-4 and PD-1 blockade reveals markers of response and resistance. Science Translational Medicine 9, eaah3560 (2017).
NRS	CLEC5A TNFSF8 LILRB2 FCGR2A CLEC7A CD86 CD14 LAIR1 ITGAM FCGR3A LILRB4 CD33 CCR1 SIGLEC9 MARCO CD163 EMILIN2 CCL8 CCL2 HOPX EFEMP1 FN1 SERPING1 SULF1 KLRC2 GZMB IL2RA TNFSF10 SERPINA1 PSTPIP2 GZMH SLAMF8 HCLS1 PDCD1LG2 TNFAIP8L2 GZMA CCL5 NKG7 CCR5 CLEC12A CST7 CXCL11 CXCL10 GBP1 LILRB1 HCST PRF1 LAG3 IFNG TNFSF14 CXCL13 SIGLEC10 IL32 CXCL9 VCAM1 CASP10 CD38 CCR2 THY1 FAP CDH11 RARRES2 COL1A1 COL3A1 COL1A2 MMP1 IL27 CLEC4E TNFSF18	Huang, A. C. et al. A single dose of neoadjuvant PD-1 blockade predicts clinical outcomes in resectable melanoma. Nat Med 25, 454–461 (2019).
chemokine	CCL2 CCL3 CCL4 CCL5 CCL8 CCL18 CCL19 CCL21 CXCL9 CXCL10 CXCL11 CXCL13	Messina, J. L. et al. 12-Chemokine gene signature identifies lymph node-like structures in melanoma: potential for patient selection for immunotherapy? Sci Rep 2, 765 (2012).
cytolytic activity	CD247 CD2 CD3E GZMH NKG7 PRF1 GZMK	Davoli, T., Uno, H., Wooten, E. C. & Elledge, S. J. Tumor aneuploidy correlates with markers of immune evasion and with reduced response to immunotherapy. Science 355, eaaf8399 (2017).

Table S2 Performance Metrics of the AIGPS-27 Model in Independent Validation Cohorts

Validation Cohort	NPV	PPV	accuracy	AUC	balanced accuracy	F1-score	recall	specificity
Hugo	0.458	0.667	0.481	0.660	0.525	0.222	0.133	0.917
Gide	0.526	0.794	0.653	0.700	0.670	0.684	0.600	0.741
Lee	0.545	0.636	0.568	0.710	0.568	0.424	0.318	0.818
MGH	0.857	0.800	0.842	0.763	0.795	0.727	0.667	0.923

Table S3 Multi-dimensional Characterization of AIGPS Gene Pairs

**Gene pair**	**Known or inferred interactions in immune or melanoma-related functions**	**Literature**	**Enriched pathways**
FCRL1-TREM1	FCRL1, primarily expressed on B cells, is associated with B cell	Scortegagna, Marzia, et al. "Age-associated	FCRL1:Cytokine Activity (GO:0005125),Receptor Ligand Activity

	activation and humoral responses. TREM1,amplifies inflammatory responses, contributes to an immunosuppressive TME. FCRL1 and TREM1 are key immune regulators with distinct, yet potentially complementary, roles within the tumor microenvironment (TME) of melanoma.	modulation of TREM1/2-expressing macrophages promotes melanoma progression and metastasis." Can cer Research (2025).	(GO:0048018);TREM1: Neutrophil Chemotaxis (GO:0030593), Neutrophil Migration (GO:1990266).
ANPEP-MS4A1	ANPEP promotes immune cell migration and MDSC infiltration. If ANPEP promotes immunosuppressive cell infiltration, it may indirectly inhibit B cell anti-tumor function. ANPEP's role in BRAF inhibitor-resistant melanoma suggests it may promote immune evasion by modulating B cells.	Griss, Johannes, et al. "B cells sustain inflammation and predict response to immune checkpoint blockade in human melanoma." Natu re communications 10.1 (2019): 4186.	ANPEP: Secretory Granule Membrane (GO:0030667), Hematopoietic cell lineage; MS4A1: Hematopoietic cell lineage.
FPR1-MS4A1	FPR1 involved in immune cell migration and inflammation, overexpression promotes melanoma cell invasion.37 FPR1, by regulating inflammatory microenvironment and cell migration, may indirectly affect MS4A1+ B cell recruitment or function.	Vo, Duong HT, Gerard McGleave, and Ian M. Overton. "Immune cell networks uncover candidate biomarkers of melanoma immunotherapy response." Journ al of Personalized Medicine 12.6 (2022): 958.	FPR1: Inflammatory Response (GO:0006954), Chemokine Receptors Bind Chemokines R-HSA-380108; MS4A1: Hematopoietic cell lineage.
LEF1-VCAM1	LEF1-driven tumor progression may lead to altered VCAM1 expression, affecting immune cell infiltration.	https://www.genecards.org/cgi-bin/carddisp.pl?gene=VCAM1	LEF1: DNA Binding, Bending (GO:0008301), Response To Cytokine (GO:0034097); VCAM1: Inflammatory Response (GO:0006954), NF-kappa B signaling pathway.
CD86-MS4A1	Immune complexes from tumor-derived IgGs (produced by B cells) stimulate CD86 expression on APCs, forming a	Griss, Johannes, et al. "B cells sustain inflammation and predict response	CD86: Receptor Ligand Activity (GO:0048018), Cellular Response To Lipopolysaccharide (GO:0071222); MS4A1:

	positive feedback loop.	to immune checkpoint blockade in human melanoma." Nature communications 10.1 (2019): 4186.	Hematopoietic cell lineage.
CD38-MS4A1	Melanoma conditioned medium induces CD38 upregulation and MS4A1 (CD20) downregulation in TAB, indicating a B cell phenotypic shift.	http://www.cancerindex.org/geneweb/MS4A1.htm	CD38: Hematopoietic cell lineage; MS4A1: Hematopoietic cell lineage.
MS4A1-TREM1	TREM1 amplifies inflammatory responses, overexpression associated with poor cancer prognosis, promotes melanoma progression/metastasis.TREM1-mediated inflammation may significantly affect local microenvironment, influencing MS4A1+ B cell recruitment, activation, and function.	Scortegagna, Marzia, et al. "Age-associated modulation of TREM1/2-expressing macrophages promotes melanoma progression and metastasis." Cancer Research (2025).	MS4A1: Hematopoietic cell lineage; TREM1: Neutrophil Chemotaxis (GO:0030593), Neutrophil Migration (GO:1990266).
MS4A1-RRM2	RRM2 is essential for DNA synthesis and cell proliferation, indirectly affecting MS4A1+ B cell proliferation.Overexpression of RRM2 in tumor cells may promote tumor proliferation, suppressing immune cells. Targeting tumor RRM2 may indirectly enhance immune response.	https://www.uniprot.org/uniprotkb/Q3THV8	MS4A1: Hematopoietic cell lineage; RRM2: Pyrimidine metabolism, G1/S-Specific Transcription R-HSA-69205.
IL1B-MS4A1	Melanoma cells release IL-1β, promoting regulatory TAB generation, directly affecting B cell phenotype and function.	Vo, Duong HT, Gerard McGleave, and Ian M. Overton. "Immune cell networks uncover candidate biomarkers of melanoma immunotherapy response." Journal of Personalized Medicine	IL1B: Cytokine Activity (GO:0005125), Inflammatory Response (GO:0006954); MS4A1: Hematopoietic cell lineage.

CXCL2-MS4A1	CXCL2, by shaping the TME's inflammatory and cellular landscape, may indirectly affect MS4A1+ B cell recruitment, localization, or functional state.	Jacquelot, Nicolas, et al. "Targeting chemokines and chemokine receptors in melanoma and other cancers." Frontiers in immunology 9 (2018): 2480.	CXCL2: Cytokine Activity (GO:0005125), Chemokine Activity (GO:0008009); MS4A1: Hematopoietic cell lineage.
